# An optimized transit peptide for effective targeting of diverse foreign proteins into chloroplasts in rice

**DOI:** 10.1038/srep46231

**Published:** 2017-04-11

**Authors:** Bo-Ran Shen, Cheng-Hua Zhu, Zhen Yao, Li-Li Cui, Jian-Jun Zhang, Cheng-Wei Yang, Zheng-Hui He, Xin-Xiang Peng

**Affiliations:** 1 State Key Laboratory for Conservation and Utilization of Subtropical Agro-bioresources, South China Agricultural University, Guangzhou, China; 2 College of Life Sciences, South China Normal University, Guangzhou, China; 3 Department of Biology, San Francisco State University, San Francisco, California, United States of America

## Abstract

Various chloroplast transit peptides (CTP) have been used to successfully target some foreign proteins into chloroplasts, but for other proteins these same CTPs have reduced localization efficiencies or fail completely. The underlying cause of the failures remains an open question, and more effective CTPs are needed. In this study, we initially observed that two *E.coli* enzymes, EcTSR and EcGCL, failed to be targeted into rice chloroplasts by the commonly-used rice rbcS transit peptide (rCTP) and were subsequently degraded. Further analyses revealed that the N-terminal unfolded region of cargo proteins is critical for their localization capability, and that a length of about 20 amino acids is required to attain the maximum localization efficiency. We considered that the unfolded region may alleviate the steric hindrance produced by the cargo protein, by functioning as a spacer to which cytosolic translocators can bind. Based on this inference, an optimized CTP, named RC2, was constructed. Analyses showed that RC2 can more effectively target diverse proteins, including EcTSR and EcGCL, into rice chloroplasts. Collectively, our results provide further insight into the mechanism of CTP-mediated chloroplastic localization, and more importantly, RC2 can be widely applied in future chloroplastic metabolic engineering, particularly for crop plants.

Chloroplasts are an important organelle in plant cells, and perform the important function of photosynthesis. Photosynthesis provides food and energy for nearly all organisms on the earth, and balances atmospheric gases by taking in CO_2_ and giving off O_2_^1^. In addition to photosynthesis, chloroplasts are capable of performing many other specialized functions, such as the assimilation of nitrate and sulphate, and the synthesis of amino acids, fatty acids, chlorophyll and carotenoids^2^. As modern biotechnology progresses, bioengineering of the metabolic pathways in chloroplasts is becoming increasingly promising, particularly as it applies to improving photosynthetic traits in crop plants. It is often necessary to precisely and efficiently introduce foreign proteins (enzymes) into chloroplasts. Currently, the most commonly-used approach is to guide the foreign protein into chloroplasts by use of a chloroplast transit peptide (CTP), which is typically fused to the N-terminus of cargo proteins. It is known that 95% of chloroplastic proteins are encoded by the nuclear genome and only a few (about 100~200) are directly encoded by the plastid genome itself 3. Nuclear-encoded chloroplastic proteins are translated in the cytoplasm as precursors, which are then imported into chloroplasts. The importation is usually mediated by a CTP in the precursor sequence, which is subsequently removed^4^.

A number of CTPs have been identified so far, and there are a number of similarities among them. CTPs commonly have a high content of hydroxylated amino acid residues (Ser, Thr, and Pro), lack acidic amino acid residues (Asp and Glu)^5^, and tend to form α-helical structures in hydrophobic environments^6^. However, there is diversity observed in their primary structures and lengths^7^. Various studies have demonstrated that CTPs guide precursor proteins through the cytoplasm into the chloroplastic stroma by interacting with certain translocators, such as 14-3-3 proteins^8^, Hsp70^9^, Hsp90^10^, FKBP^11^ and the TOC and TIC complexes, which are located at the outer and inner envelopes of chloroplasts, respectively^3^,^12^.

In 1985 the foreign protein NPT-II (bacterial neomycin phosphotransferase II) was successfully transported into tobacco chloroplasts by linking a 58-amino acid pea rubisco small subunit (rbcS) transit peptide to the N-terminus^13^. Since that time, several CTP sequences have been identified from various chloroplast proteins, such as chlorophyll a/b-binding protein^14^, ferredoxin^15^, and granule-bound starch synthase^16^. These CTPs were all able to target certain foreign proteins into chloroplasts, but the rbcS CTP is the most commonly used and is considered to be the most efficient^17^,^18^,^19^. However, problems and failures with the use of these CTPs have been reported. For example, BMV (the coat protein of brome mosaic virus) was inefficiently targeted into chloroplasts by a soybean rbcS CTP *in vitro*^20^, and N-acetyltransferase from sheep failed to be targeted into rice chloroplasts by a rice CTP *in vivo*^21^. It has also been reported that a portion of the mature protein following the transit peptide is required for importing some foreign proteins into chloroplasts^14^,^22^,^23^. For example, translocating EPSP (5-enolpyruvyl-3-phosphoshikimate synthase) into chloroplasts required the pea rbcS transit peptide together with the adjacent 24 amino acids of the mature protein^22^. While most proteins do not necessarily need this additional portion for their localization^18^,^24^,^25^,^26^. Several reports have noted that sequences following the CTP may also mediate the translocation of precursor proteins, however, the real function and mechanism of this portion remains to be clarified^27^,^28^,^29^,^30^.

Through the use of modern biotechnology, a wide range of metabolic engineering work is being conducted in plants; work which aims to make improvements in photosynthesis, stress resistance, grain quality, yield, etc. Metabolic engineering work is still limited for rice, despite the importance of rice as a staple food crop. For the engineering process, it is commonly necessary to localize specific functional foreign proteins into rice chloroplasts, and thus a highly efficient CTP is crucial. In this study we initially attempted to introduce two bacterial enzymes (EcGCL: *E.coli* glyoxylate carboligase; EcTSR: *E.coli* tartronic semialdehyde reductase) into rice chloroplasts, in order to construct a photorespiratory bypass within chloroplasts. Unexpectedly, we found that the rice rbcS CTP (rCTP) failed to localize these two bacterial enzyme proteins to rice chloroplasts, and the enzymes were subsequently degraded. Therefore, we then investigated the cause of the localization failure. Our results revealed that an unfolded region between rCTP and its cargo protein is critical for chloroplastic targeting. We optimized the rCTP sequence so that it was able to more effectively import diverse proteins into rice chloroplasts.

## Results

### EcTSR and EcGCL failed to be targeted into rice chloroplasts by rCTP and were subsequently degraded

**Figure 1:**
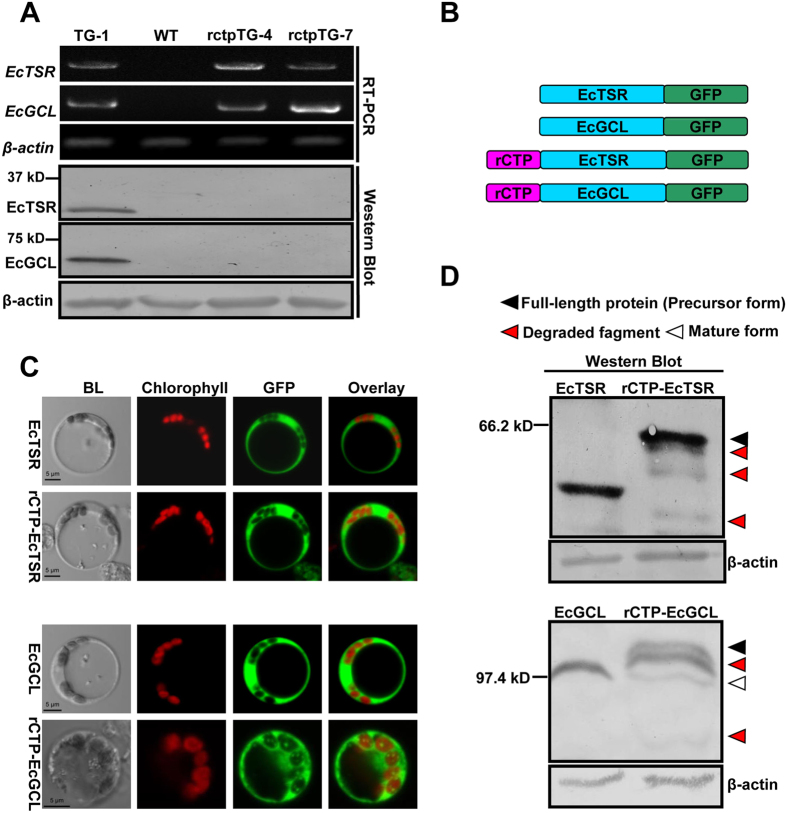
Expression analyses of *EcTSR* and *EcGCL* as targeted by rCTP in either rice transgenic plants or protoplasts. (**A**) Total RNA extracted from leaves of wild type (WT) and three transgenic lines (TG-1, rctpTG-4 and rctpTG-7) was used for RT-PCR analysis using primers specific for *EcTSR* and *EcGCL*. The rice *β-actin* gene was used as a control. The corresponding total protein extracts were analyzed by Western Blot using EcTSR and EcGCL polyclonal antibodies, and β-actin protein was probed by using a monoclonal β-actin antibody as loading control. The theoretical molecular weight of EcTSR, rCTP-EcTSR, EcGCL and rCTP-EcGCL is about 31 kD, 36 kD, 65 kD and 70 kD, respectively. (**B**) Structures of the fusion genes. (**C**) Plasmids containing the *EcTSR-GFP* (**EcTSR**), *rCTP-EcTSR-GFP* (**rCTP-EcTSR**), *EcGCL-GFP* (**EcGCL**), or *rCTP-EcGCL-GFP* (**rCTP-EcGCL**) fusion genes were introduced into rice protoplasts. Cells are imaged by a confocal microscope at 24 h after the transformation. **BL**, bright light; **chlorophyll**, chloroplast chlorophyll autofluorescence; **GFP**, GFP fluorescence. **(D)** Proteins extracted from the transformed protoplasts were analyzed by Western Blot using a monoclonal anti-GFP antibody, and β-actin protein was probed by using a monoclonal β-actin antibody as loading control. **EcTSR**, protein extracts from *EcTSR*-*GFP* transformed protoplasts; **rCTP-EcTSR**, from *rCTP*-*EcTSR*-*GFP*; **EcGCL**, from *EcGCL*-*GFP*; **rCTP-EcGCL**, from *rCTP-EcGCL*-*GFP*. The triangles in black, white or red represent the precursor form, mature form or degraded fragment respectively.

It was previously reported that when the bacterial-type glycolate pathway was introduced into the chloroplasts of Arabidopsis, both photosynthesis and biomass were improved^31^. We attempted to introduce the same pathway into rice, in order to apply this approach in a plant with more practical significance. The rice rubisco small subunit (rbcS) transit peptide (rCTP) was firstly used as the chloroplast transit peptide since it was shown to be able to efficiently direct foreign proteins such as GFP^26^ (Supplemental Fig. S1) and cry1Ac^18^ into chloroplasts in the transgenic rice plants. The *rCTP* sequence was fused with the two *E.coli* genes *glyoxylate carboligase (EcGCL*) and *tartronic semialdehyde reductase (EcTSR*), and then cloned into the multiple genes expression vector pYL1305. The constructed vector (named rctpTG-pYL1305) and a control vector (named TG-pYL1305) were transformed into rice (Structure of the vectors is shown in Supplemental Fig. S2). RT-PCR analysis showed that the target genes were expressed abundantly at the mRNA level in both types of transgenic plants. Protein expression could be detected in the TG-pYL1305 line TG-1, but not in the rctpTG-pYL1305 lines rctpTG-4 and rctpTG-7 (Fig. 1A). This result indicated that it was the fusion of EcGCL or EcTSR with rCTP that resulted in the failure to accumulate the target proteins in the transgenic rice plants.

To determine how *rCTP* affects the expression of *EcTSR* and *EcGCL, EcTSR-GFP* or *EcGCL-GFP* fused with or without *rCTP* (Fig. 1B) were transfected into rice protoplasts for transient expression analysis. Confocal observation demonstrated that all EcTSR-GFP, rCTP-EcTSR-GFP and EcGCL-GFP protein, and most rCTP-EcGCL-GFP protein, were retained in the cytoplasm (Fig. 1C). Through Western Blot analysis, protein fragments with lower molecular mass than the native protein, which is a typical characteristic of protein degradation^32^, were detected for the rCTP fusion proteins, but not for the proteins without rCTP (Fig. 1D). This result suggests that rCTP failed to localize EcTSR and EcGCL to chloroplasts, and that some of the precursor proteins were subsequently degraded. This degradation is likely the reason that the precursor proteins could not be detected in the transgenic rice plants harboring *rCTP*-*EcTSR* and *rCTP*-*EcGCL* (Fig. 1A).

### Targeting capability of rCTP depends on its cargo protein

**Figure 2:**
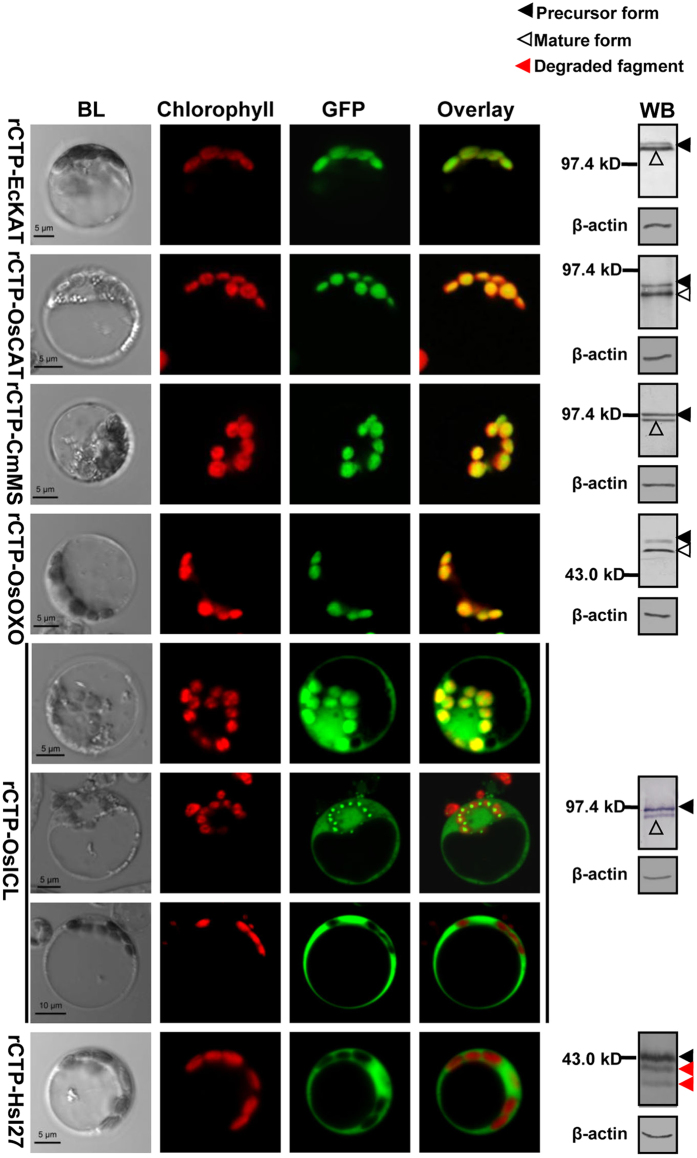
Subcellular localization of various rCTP fusion proteins. Plasmids containing fusion genes of *rCTP-EcKAT-GFP* (**rCTP-EcKAT**), *rCTP-OsCAT-GFP* (**rCTP-OsCAT**), *rCTP-CmMS-GFP* (**rCTP-CmMS**), *rCTP-OsOXO-GFP* (**rCTP-OsOXO**), *rCTP-OsICL-GFP* (**rCTP-OsICL**) or *rCTP-HsI27-GFP* (**rCTP-HsI27**) were introduced into rice protoplasts. **WB**, Western Blot. Other legends are the same as described in Fig. 1.

We further tested the targeting capability of rCTP for six additional cargo proteins (Fig. 2): EcKAT (*E.coli* katalase); HsI27 (the 27th Ig domain of the human muscle protein titin, a remarkably stable folded artificial passenger, which is usually used to analyze the unfolded properties of the mitochondrial and chloroplast translocation); CmMS (cucumber malate synthase); OsCAT (rice catalase); OsOXO (rice oxalate oxidase); and OsICL (rice isocitrate lyase). The natural targeting sequences of the cargo proteins, if any, were removed before rCTP was linked to the N-terminus. Protoplast transient expression analysis demonstrated that EcKAT, OsCAT, CmMS, OsOXO could be efficiently localized to chloroplasts by rCTP. In contrast, rCTP was completely unable to guide HsI27 into rice chloroplasts. Three different patterns were observed for OsICL: (i) evenly distributed in both cytoplasm and chloroplasts; (ii) mostly in the cytoplasm; or (iii) completely in the cytoplasm. This result suggests that OsICL was targeted to chloroplasts less efficiently by rCTP comparing with the other four proteins during the transient expression assay. This notion was further verified by Western Blot analysis, since large amount of OsICL proteins was detected as precursor forms. Similar to EcTSR, protein fragments were detected for HsI27 (Figs 1D vs. 2), indicating that this protein also failed to be imported into chloroplasts and was subsequently degraded.

### Tertiary structure of EcTSR affects the targeting capability of rCTP

**Figure 3:**
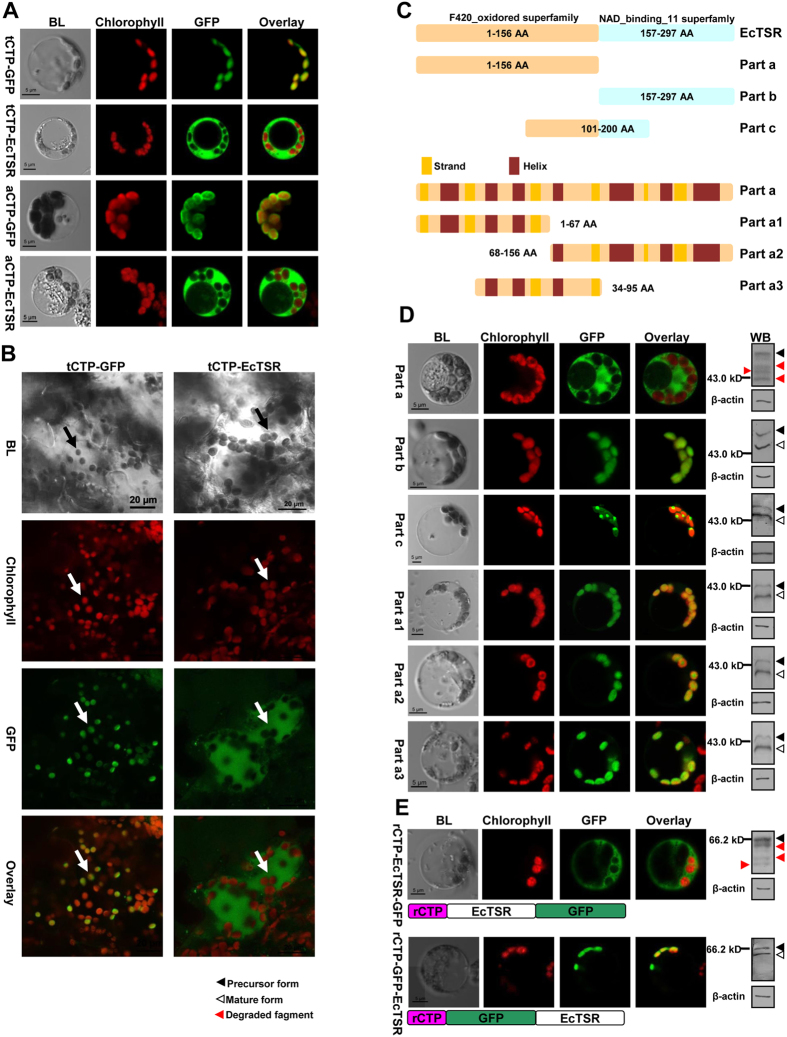
The targeting capability of rCTP in relation to the tertiary structure of EcTSR. **(A**) Plasmids containing the *tCTP-GFP* (**tCTP-GFP**), *tCTP-EcTSR-GFP* (**tCTP-EcTSR**), *aCTP-GFP* (**aCTP-GFP**) or *aCTP-EcTSR-GFP* (**aCTP-EcTSR**) fusion genes were introduced into rice protoplasts. **(B**) Plasmids containing the *tCTP-GFP* (**tCTP-GFP**) or *tCTP-EcTSR-GFP* (**tCTP-EcTSR**) fusion genes were transformed into tobacco leaves via Agrobacterium. Mesophyll cells were imaged by a confocal microscope at 48 h after the transformation. Arrows direct the position of chloroplasts. **(C**) EcTSR was divided into 6 part, part a (from amino acids 1 to 156), part b (157 to 293), part c (101 to 200), part a1 (1 to 67), part a2 (68 to 156) and part a3 (34 to 95), each part was fused with rCTP and GFP for transient expression analysis. **(D**) Plasmids containing the part a, part b, part c, part a1, part a2 or part a3 fusion genes were introduced into rice protoplasts. Proteins extracted from the transformed protoplasts were analyzed by Western Blot. **(E**) Plasmids containing the *rCTP-EcTSR-GFP* or *rCTP-GFP-EcTSR* fusion genes were introduced into rice protoplasts. Proteins extracted from the transformed protoplasts were analyzed by Western Blot. Other legends are the same as described in Fig. 1.

To determine whether rbcS CTP sequences from different species have different localization capabilities, tobacco rbcS chloroplast transit peptide (tCTP) and Arabidopsis chloroplast rbcS transit peptide (aCTP) were used in place of rCTP to guide EcTSR into chloroplasts in rice protoplasts and tobacco leaves. As for rCTP, we observed that aCTP and tCTP failed to localize EcTSR to the chloroplasts in rice and tobacco (Fig. 3A,B). This result suggests that the cargo protein EcTSR sequence is incompatible with the rbcS CTPs.

To determine how the cargo protein EcTSR affects localization we divided the EcTSR sequence into three parts. Part a, from amino acids 1 to 156, contains the first domain, which belongs to the F420 oxidoreductase superfamily; part b, from amino acids 157 to 293, contains the second domain, which belongs to the NAD binding 11 superfamily; and part c, from amino acids 101 to 200, overlaps with part a and part b (Fig. 3C). Each part was fused with rCTP at the N-terminus and with GFP at the C-terminus and transfected into rice protoplasts. Part b and part c were successfully targeted to the chloroplast, but part a was not and degraded, suggesting that the cause of the failure is in part a (Fig. 3D). *In silico* analysis showed that there are 13 secondary structures in part a (Supplemental Fig. S3A). We further subdivided the part a sequence into three parts (a1, a2 and a3), with none of our subdivision breakpoints occurring within a secondary structural element, *i.e.*, secondary structures 1–6 are entirely in a1, 4–7 in a2, and 7–13 in a3 (Fig. 3C). Transient expression analysis showed that a1, a2 and a3 could all be properly localized to chloroplasts by rCTP (Fig. 3D). These results suggest that the cause of the localization failure is not in the primary sequence or individual secondary structures, and is therefore likely caused by the entire tertiary structure of part a being next to rCTP (the tertiary structure of part a is shown in Supplemental Fig. S3B). To further test this idea, we constructed a fusion gene in which *GFP* was inserted between *rCTP* and *EcTSR* sequences, *i.e., rCTP-GFP-EcTSR*. As predicted, this change allowed EcTSR to be properly localized to rice chloroplasts, in sharp contrast to *rCTP-EcTSR-GFP* (Fig. 3E). The above results collectively suggest that the tertiary structure of EcTSR spatially affects the function of rCTP.

### The N-terminal unfolded region of cargo proteins determines the targeting capability and efficiency of rCTP

**Figure 4:**
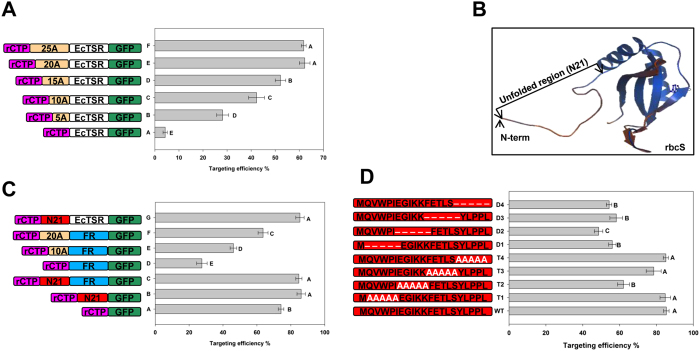
The targeting efficiency of rCTP for EcTSR in relation to the N-terminal unfolded region. **(A**) Different numbers of alanine residues (5, 10, 15, 20, 25) were introduced into the N-terminus of the EcTSR protein for chloroplast targeting efficiency analysis. **(B**) Tertiary structure of the rice rbcS mature protein. **(C**) The N21 of rbcS was removed or replaced by 10 or 20 alanine residues for chloroplast targeting efficiency analysis. **(D**) The N21 mutants (T1 through T4, D1 through D4) together with the rCTP were fused with EcTSR-GFP and introduced into rice protoplasts to calculate the chloroplast targeting efficiency. The data represent means ± SD of three biological replicates (*n* = 3). Different capital letters in the same column indicate significant differences at P < 0.01 according to Duncan’s multiple range test.

In order to determine why some proteins failed to be localized properly, we analyzed the tertiary structure of the eight proteins used in this report. We found that EcTSR, HsI27 and EcGCL, which failed to be properly localized by rCTP, have a common feature in that there are few amino acid residues in the N-terminal unfolded region (N-terminal region without secondary structure or tertiary structure in the PDB or SWISS-MODEL structures). By analyzing the number of amino acids in this region and comparing it with targeting efficiency, we found that a positive correlation existed between them (Table 1). To experimentally test this relationship, we introduced different numbers of alanine residues into the N-terminus of EcTSR to make an artificial unfolded region. The results demonstrated that targeting efficiency increased as the number of alanines increased, and reached a maximal efficiency (about 60%) at 20 alanines (Fig. 4A).

We further analyzed the structures of the mature rbcS proteins from rice, tobacco, pea and Arabidopsis, and found that all of these proteins contain a 21 amino acid unfolded region (N21) in the N-terminus (Fig. 4B, Supplemental Fig. S4). To further understand the function of N21, we divided the rice rbcS precursor into three parts: (i) rCTP; (ii) N21 (21 amino acid unfolded region); and (iii) FR (folded region). Removing the N21 region from the rbcS protein decreased its localization efficiency from 85.7% to 27.5%. The decreased efficiency recovered from 27.5% to 46.1% or 63.6% when a 10 or 20 alanine fragment was inserted in place of N21, respectively. Further, when we linked the N21 sequence with EcTSR, the targeting efficiency of EcTSR was increased from 4.7% to 85.3%, similar to that of rbcS (Fig. 4C). All together, these results suggest that the length of the unfolded region of the cargo protein determines the chloroplastic targeting capability and efficiency of rCTP, with about 20 amino acids providing maximal efficiency.

As described above, the addition of a 20-alanine sequence to rCTP increased EcTSR targeting efficiency from 4.7% to about 60%, but this value is still lower than the 85% observed when the N21 sequence was used (Fig. 4A vs. C). To determine which N21 amino acid residues are likely responsible for increasing targeting efficiency, we generated various mutated N21 sequences (every five-amino-acid fragment in N21 was replaced by five alanines or deleted), and fused them with EcTSR to assay their targeting efficiency. The targeting efficiency of the T2 mutant, in which the EGIKK sequence was replaced by five alanines, was decreased to 62.1% from 85.3%, suggesting that the EGIKK residues play a functional role in targeting (Fig. 4D). The targeting efficiency of deletion mutants were all decreased to about 55%, further supporting the idea that an unfolded sequence of at least 20 amino acids is required for efficient chloroplastic localization.

To further explore if an unfolded region behind CTP is common for nuclear-encoded chloroplast proteins, we collected 80 chloroplast precursor protein sequences with known tertiary structures from the PDB database. The lengths of CTPs and the N-terminal unfolded regions in mature proteins for these 80 precursor proteins were analyzed *in silico*. 37 of the proteins contain an unfolded region longer than 20 amino acids in the N-terminus of their mature proteins (Supplemental Table S1). More interestingly, we noticed that a shorter CTP sequence always occurs with a longer unfolded sequence, and vice versa (Supplemental Fig. S5). We estimated that a 70 amino-acid length is required for a CTP to have high targeting competence, based on our observations that a 20 amino acid unfolded sequence is required for EcTSR to be efficiently targeted into chloroplasts by a 48 amino-acid rCTP. By analyzing the 80 precursor proteins mentioned above, the average length of their CTPs plus the corresponding unfolded regions was 76 amino acids.

### Generation of an optimized rCTP and its performance in transgenic rice plants

**Figure 5:**
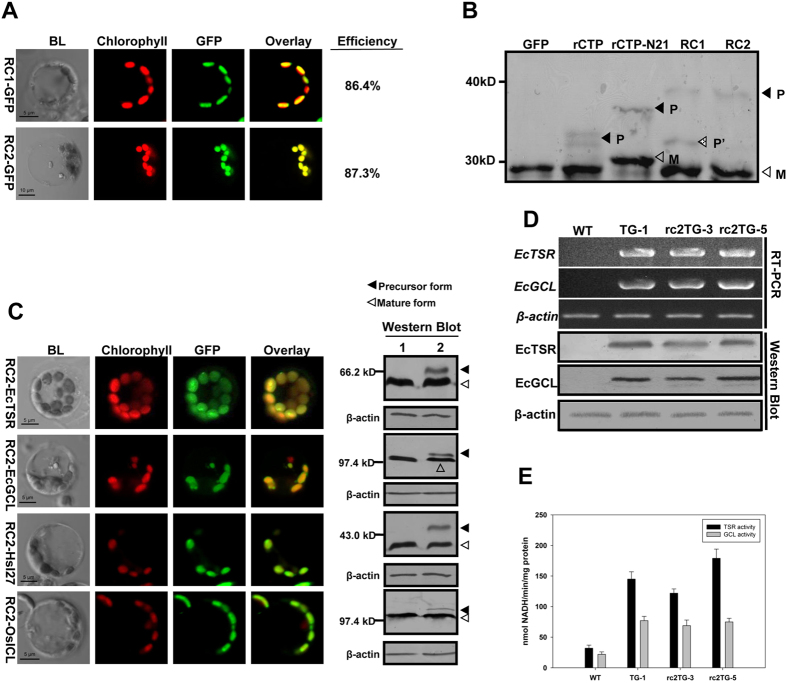
Generation of an improved rCTP and its performance in transgenic rice plants. **(A)** Plasmids containing the *RC1-GFP* and *RC2-GFP* fusion genes were introduced into rice protoplasts to calculate the chloroplast targeting efficiency. **(B)** Plasmids containing the *GFP* gene or *rCTP-GFP, rCTP-N21-GFP, RC1-GFP, RC2-GFP* fusion genes were introduced into rice protoplasts. Proteins extracted from the transformed protoplasts were analyzed by Western Blot. **GFP**, protein extracts from *GFP* transformed protoplasts; **rCTP**, from *rCTP-GFP*; **rCTP-N21**, from *rCTP-N21-GFP*; **RC1** and **RC2**, from *RC1-GFP* and *RC2-GFP*; **P**, Precursor form; **P’**, intermediate form, in which rCTP was removed but N21 and BS remained at the N-terminus of GFP protein; **M**, mature form. **(C)** Plasmids containing RC2 fusion genes (*RC2-EcTSR-GFP, RC2-EcGCL-GFP, RC2-OsICL-GFP, RC2-HsI27-GFP*) and genes without RC2 (*EcTSR-GFP, EcGCL-GFP, OsICL-GFP, HsI27-GFP*) were introduced into rice protoplasts. Protein extracts from transformed protoplasts were analyzed by Western Blot. **1**, Protein extracts from control samples; **2**, Protein extracts from RC2 fusion proteins samples. **(D)** Total RNA and protein were extracted from leaves of wild type (WT) and the three transgenic lines (TG-1, rc2TG-3 and rc2TG-5) and analyzed by RT-PCR and Western Blotting. **(E)** Enzyme activities of EcTSR and EcGCL in leaves of wild type (WT) and three transgenic lines (TG-1, rc2TG-3 and rc2TG-5). The data represent means ± SD of three biological replicates (*n* = 3). Other legends are the same as described in Fig. 1.

Comparative analyses of rCTP, tCTP, and aCTP with their own N21 regions showed that rCTP-N21 was always the best at guiding either GFP or EcTSR into rice chloroplasts (Supplemental Fig. S6). Thus, we started with rCTP-N21 as a basal template and sought to further optimize its efficiency. The added N21 sequence must be removed after importation, otherwise enzyme activities would be affected. Various CTPs for nuclear-encoded chloroplast proteins are typically removed by a stromal processing peptidase (SPP)^4^. The binding and cutting sequences for SPP have been identified as BS1 from the *Silene pratensis* plastocyanin precursor^4^ or BS2, which we identified from the rice rbcS precursor based on Richter’s theory^4^ (Supplemental Fig. S7A). We linked either the BS1 or the BS2 sequence to the C-terminus of rCTP-N21 (Supplemental Fig. S7B), and named them here as RC1 and RC2, respectively. The addition of BS1 or BS2 had no negative effects on the targeting efficiency (Fig. 5A). To test whether the introduced BS can be correctly recognized and subsequently cut by SSP after importation, Western Blot analysis was carried out using proteins extracted from transfected protoplasts. We observed that RC1 and RC2 could be properly removed after importation, with RC2 being more efficiently removed than RC1 (Fig. 5B).

We thus chose RC2 as an optimized CTP to further test its targeting capability and efficiency for EcTSR, EcGCL, HsI27 and OsICL. Protoplast transient expression analysis showed that RC2 was much more effective than rCTP at localizing the four proteins. The targeting efficiency was up to 85% for all four proteins, and the RC2 sequence was completely removed after importation into chloroplasts (Fig. 5C, Supplemental Table S4).

Determining how well RC2 functions in rice plants must be clearly defined, so that it can be used in practical applications. To determine this, we constructed a plasmid containing the *RC2-EcTSR* and *RC2-EcGCL* fusion genes (named rc2TG-pYL1305, Supplemental Fig. S2), and then genetically transformed it into rice. The *EcTSR* and *EcGCL* fusion genes were abundantly expressed at the levels of mRNA, protein and enzyme activity in the rc2TG-pYL1305 transgenic rice (Fig. 5D and E), in sharp contrast to the results from the rctpTG-pYL1305 transgenic rice (Fig. 1A). Furthermore, Western Blot analysis showed that the molecular sizes of the EcTSR and EcGCL proteins in the rc2TG-pYL1305 transgenic lines were the same as those in the control TG-pYL1305 transgenic line TG-1 (Fig. 5D), indicating that RC2 was completely removed after EcTSR and EcGCL were targeted into rice chloroplasts.

## Discussion

Failure to express foreign proteins has frequently been observed in transgenic plants, and can have a number of different causes, such as positional effects of the local chromatin environment^33^, homology-dependent gene silencing^34^, codon preference^35^, and protein degradation^32^. In this study, we report that two bacterial genes, *EcTSR* and *EcGCL*, which were fused with *rCTP*, could be expressed at the mRNA level but not at the protein level in rice (Fig. 1A). Protoplast transient expression analysis demonstrated that the two fusion proteins accumulated in the cytoplasm (Fig. 1C). In plants, the flux of protein import into chloroplasts is high and also varies widely, and cytosolic precursor proteins are seldom observed, suggesting that there is an efficient mechanism to control precursor protein levels in the cytoplasm^36^. To prevent accumulation of the precursor proteins in the cytoplasm, they often associate with cytosolic chaperones, such as 14-3-3 and Hsp70, for highly efficient targeting, and their synthesis is regulated by retrograde signals from the plastid to the nucleus^37^. In spite of this, precursor proteins may still be inefficiently imported and remain in the cytoplasm, especially when their expression levels exceed the import capacity^37^. These un-importable precursor proteins in the cytoplasm will be degraded by the ubiquitin-proteasome system (UPS). It was documented that the degradation of un-imported precursor proteins in the cytoplasm is mediated by a cytosolic Hsp70 subfamily isoform, Hsc70-4, and a cytosolic E3 ubiquitin ligase, CHIP. Hsc70-4 recognizes and binds to the transit peptide, and CHIP subsequently ubiquitinates Hsc70-4 bound proteins for degradation^38^. Two recognition sequences for Hsc70-4 were identified in the Arabidopsis rbcS transit peptide^38^. The Hsc70-4 recognition sequences may also exist in rCTP, thus it is highly possible that the rCTP fusion proteins would be degraded if they remained in the cytoplasm. This notion was verified by our current study (Fig. 1D), as degradation occurred for the un-imported EcTSR and EcGCL to such an extent that no precursor protein could be detected in those transgenic rice plants.

The mature part of a protein may play an important role in determining the targeting competence of chloroplast precursors^3^. It was proposed that the positively charged amino acids following the transit peptide could interact with the negatively charged lipids of the chloroplast membrane^39^, and that the C-terminus of the pea rbcS precursor modulated its interaction with the translocation apparatus and PIRAC (protein import related anion channel)^29^. These relevant studies focused primarily on native precursor proteins, therefore, how foreign proteins affect translocation remains to be further understood. In this study, we demonstrated that the targeting efficiency of rCTP varied when used with different cargo proteins. Some proteins, such as EcTSR and HsI27, were totally unable to be targeted into rice chloroplasts by rCTP (Figs 1 and 2). Further analysis indicated that the entire F420 domain of EcTSR, but not its primary sequence or secondary structures, affects the efficiency of translocation (Fig. 3C and D; Supplemental Fig. S3). Furthermore, efficient transport could be restored by separating rCTP from EcTSR by GFP (Fig. 3E), suggesting that the tertiary structure of EcTSR spatially affects the function of rCTP. The chloroplast translocation process can be divided into three steps: (i) the precursor navigates through the cytoplasm to the chloroplastic outer envelope membrane; (ii) the precursor is transported across the outer and inner envelope membrane; and (iii) the transit peptide of the transported precursor is cleaved off by SPP (stromal processing peptidase) in the stroma, and the mature protein released^3^. Our expression analyses showed that EcTSR and HsI27 fusion proteins were evenly distributed in the cytoplasm (Figs 1 and 2), without surrounding the chloroplast, suggesting that translocation is disabled during the first step of targeting. Although it is still unclear whether any specific cytosolic translocators exist for protein navigation through the cytoplasm to the chloroplastic outer envelope membrane, several proteins that interact with transit peptides have been identified^37^. Analysis by an *in vitro* import system has shown that precursors interact with 14-3-3 and Hsp70 to form a complex, and this complex is more efficiently imported into chloroplasts compared to the precursors alone^8^. It is accepted that precursors have to be unfolded at the chloroplast surface prior to importation across envelope membranes^37^. However, it has recently been reported that foreign proteins, such as HsI27, need to be folded before binding to the translocons at the chloroplast surface. Moreover, in contrast to the native precursor protein, HsI27 fusion proteins did not interact with Hsp70^40^. Similar to EcTSR, HsI27 was also unable to be targeted into chloroplasts by rCTP. Taken together, the results suggest that foreign proteins may be folded prior to translocation, and thus, the tertiary structure of cargo proteins would influence the interaction of rCTP with cytosolic translocators so as to affect the targeting efficiency.

Comai *et al*. reported earlier that a 23 amino acid sequence in the pea mature rbcS was required for the chloroplastic translocation of the EPSP protein. The authors interpreted that some features in this region may be important for the transport process, or this sequence may function as a spacer region^22^. It has been proved that this 23 amino acid region possessed the information for translocation^28^. Our data also showed that the EGIKK fragment in this region may play a functional role in enhancing targeting efficiency (Fig. 4D). However, in most cases this region is not necessarily needed for the chloroplastic targeting function, such as for NPT-II^13^ and cry1Ac^18^. Thus, the real function and mechanism of this region remains to be addressed. In this study, we support Comai’s later idea that there exists an unfolded region that may function as a spacer, and suggest that a length of about 20 amino acids for this region is optimal for chloroplastic targeting. This is based on the following evidences: (i) the un-importable proteins in this study or former studies (EcTSR, EcGCL, HsI27, EPSP) have a common feature of less than three amino acids in the unfolded region, whereas the importable proteins usually have more than 18 amino acids in this region (Table 1); (ii) the rbcS mature protein from rice, tobacco, pea and Arabidopsis all contains a 21 amino-acid unfolded sequence (N21), which was shown to be crucial for rbcS translocation (Fig. 4B and C; Supplemental Fig. S4); and (iii) inserting an unfolded sequence (20 alanines or N21) between rCTP and EcTSR markedly improved chloroplastic targeting efficiency (Fig. 4A).

Steric hindrance caused by protein tertiary structure commonly exists for protein-protein interaction, and may influence the function of proteins. During generation of a biofunctional fusion protein by bioengineering, steric hindrance was usually alleviated by inserting a spacer (usually 5 to 20 amino acids) between the two target proteins^41^. Since the tertiary structure of cargo proteins may spatially influence the function of rCTP as described above, we suggest that the unfolded region can function as a spacer to separate rCTP from cargo proteins. This spacer alleviates the steric hindrance, such that rCTP can be more accessible to binding certain translocators. Such a proposed spacer is common for natural nuclear-encoded chloroplast proteins, since 37 of the 80 studied proteins contain an unfolded region longer than 20 amino acids in the N-terminus of their mature proteins (Supplemental Table S1). In silico analysis also showed that a shorter CTP sequence always occurs with a longer unfolded sequence, and vice versa (Supplemental Fig. S5). The length of CTPs varies strongly, from 10 to 150 amino acids^2^. By comparing the targeting efficiency of different length of CTPs, Bionda *et al*. noted that pOE33 CTP with 76 amino acids could direct HsI27 into tomato chloroplasts, but CTPs of pRbl11 and pNTT1 with 49 and 21 amino acids, respectively, could not. Extending the pNTT1 CTP to 60 amino acids by addition of its following sequence in mature domain allowed HsI27 to be targeted. The authors suggested that about 60 amino acids length of N-terminal sequence remaining unfolded is required for efficiently targeting proteins into chloropalsts^23^. Our current study supported their suggestion, and further raised a mechanistic notion that a longer CTP may spare space so as to be less influenced by its cargo proteins. If the CTP is too short, addition of an unfolded spacer may alleviate the spatial hindrance. Based on our data, it is estimated that the combined length of transit peptide and the N-terminal unfolded region of cargo proteins is about 70 amino acids in rice.

The rbcS transit peptide is the most commonly used CTP for targeting foreign proteins into chloroplasts^17^,^19^,^42^. However, problems still exist with this CTP, especially when it is used for proteins with an unfolded region that is less than 20 amino acids in length. We also showed evidence that inserting an N21 region can guarantee that more diverse foreign proteins can be efficiently imported into rice chloroplasts. Finally, we constructed a chimeric transit peptide that is composed of the rice rbcS transit peptide, the N21 region and the extra cleavage site BS2. This optimized CTP named RC2 (the nucleic acid sequence and amino acid sequence were shown in Supplemental Fig. S7C) was shown to greatly improve the chloroplastic localization for EcTSR and EcGCL, and was able to more effectively target diverse foreign proteins into rice chloroplasts. Therefore, RC2 can be applied in future chloroplastic metabolic engineering, particularly for crop plants.

## Materials and Methods

### Plant Materials and growth conditions

*Oryza sativa* cv. Zhonghua 11 (japonica cultivar-group) was used for constructing the transgenic lines and for protoplast isolation. *Nicotiana tabacum* cv. Zhongyan 90 was also used in the transient expression analysis.

Pre-germinated seeds of rice were grown in Kimura B complete nutrient solution^43^ in a greenhouse condition with a cycle of 14 h light/10 h dark (30/25 °C) at 800–1000 μmol photons m^−2^ s^−1^, relative humidity 60–80%. Tobacco were grown in soil in a greenhouse condition with a cycle of 14 h light/10 h dark (25/21 °C) at 400–1000 μmol photons m^−2^ s^−1^, relative humidity 60–80%.

### Plasmid construction

DNA sequences, which encode the rbcS from rice (Os12g0274700), tobacco (NP_001295873.1) and Arabidopsis (AT1G67090), the chloroplast transit peptides, the N21, the mutated N21, the BS1 and BS2, the GFP, and the eight studied proteins, *i.e.*, EcTSR (WP_021571777.1), EcGCL (WP_061352092.1), EcKAT (WP_000077872.1), OsCAT (Os03g0131200), OsICL (Os07g0529000), OsOXO (Os03g0693900), CmMS (CAA40262.1), HsI27 (NP_001254479.2), were amplified by polymerase chain reaction (PCR), with the *in situ* peroxisomal targeting sequences being removed for OsCAT, OsICL and CmMS. The primers and the templates used for PCR were listed in Supplemental Table S2, the restriction enzymes used for generating the fusion genes were listed in Supplemental Table S3.

For rice protoplast transient expression, fusion genes were introduced into pYL322-DI, under the control of the CaMV 35S promoter. To generate the tCTP-GFP-pBI121 and tCTP-EcTSR-GFP-pBI121 vectors for tobacco leaves transient expression, the *GUS* gene sequence in pBI121 was replaced by the *tCTP-GFP* and *tCTP-EcTSR-GFP* fusion gene sequences. To generate the TG-pYL1305, rctpTG-pYL1305, rc2TG-pYL1305 vectors for transgenic rice plants, the *EcTSR* and *EcGCL* genes expression cassettes were introduced into the multiple genes expression vector pYL1305 by homologous recombination method^44^. To construct the vectors for protein expression in *E. coli*, complete *EcTSR* or *EcGCL* genes sequences were introduced into the bacterial expression vector pCold™ IV (TaKaRa, Japan). The pYL322-DI and the pYL1305 vectors were kindly provided by Dr Yao-Guang Liu, College of Life Sciences, South China Agricultural University.

### Generation of transgenic rice

The constructed vectors (TG-pYL1305, rctpTG-pYL1305 and rc2TG-pYL1305) were introduced into rice by *Agrobacterium*-mediated infection (strain EHA105)^45^. To screen the positive T_0_ lines, hygromycin-resistance gene (HPT) was detected by PCR using the total rice leaves DNA as template. The primers for HPT PCR detection were as follows: 5′-CTGAACTCACCGCGACGTCTGTC-3′ and 5′-TAGCGCGTCTGCTGCTCCATACA-3′. Total rice leaves DNA was extracted by TaKaRa plant DNA isolation reagent (TaKaRa, Japan).

The seeds harvested from the positive independent T_0_ lines (TG-1, rctpTG-4 and rctpTG-7, rc2TG-3 and rc2TG-5) were germinated in complete Kimura B nutrient solution and then transferred to soil to grow for the subsequent analysis.

### Transient expression in rice protoplast and tobacco

The rice protoplasts were isolated according to Zhang *et al*.^46^. For protoplast transformation, 10 μg of constructed plasmid was transfected into 100 μL of protoplasts (about 2.0 × 10^5^ cells) by PEG treatment as described previously^46^. After that, the protoplasts were incubated under dark at 25 °C for 24 h. For tobacco transient expression, intact young tobacco leaves were transfected by Agrobacterium strains LBA4404, which harbor the transient expression vectors, and were incubated under dark at 25 °C for 48 h as described previously^47^. The confocal images were captured using the ZEISS LSCM 780 system. The excitation wavelength for GFP green fluorescence and chlorophyll autofluorescence was 488 nm. The emission wavelength for GFP green fluorescence was 493–546 nm, the emission wavelength for chlorophyll autofluorescence was 658–735 nm.

### Protein expression, purification and antibody preparation

*E. coli* BL21 (DE3) was used for bacterial expression of EcTSR and EcGCL. Protein expression was induced with 1 mM IPTG, after incubation for 24 h at 16 °C, cells were harvested by centrifugation at 4000 rpm for 10 min at 4 °C, washed and resuspended in 50 mM PBS (pH 8.5) containing 500 mM NaCl and 5% glycerol, then ruptured on ice by ultrasonic for 10 min. Cell debris was removed by centrifugation at 12000 rpm for 20 min at 4 °C, and the supernatant was loaded onto a nickel chelating affinity chromatography column previously equilibrated with 50 mM PBS (pH 7.8) containing 500 mM NaCl, 5 mM imidazole and 5% glycerol, then washed with 20 column volumes of 50 mM PBS (pH 7.8) containing 500 mM NaCl, 10 mM imidazole and 5% glycerol. The enzymes were eluted with 5 column volumes of 50 mM PBS (pH 7.8) containing 300 mM NaCl, 150 mM imidazole and 5% glycerol. The purification fractions were desalted by ultrafiltration and purity was checked by SDS-PAGE. The rabbit polyclonal antibody for EcTSR or EcGCL was prepared by Sangon (Sangon, China) using the corresponding purified protein as antigen.

### RNA isolation and RT-PCR

Total RNA was extracted from rice leaves using TRIzol reagent (Life Technologies, USA), and treated with RNase-free DNaseI (Amersham, USA). The quality and quantity of the purified RNA was assessed with a NanoDrop-1000 (NanoDrop, USA). First-strand cDNA was synthesized using ReverTra Ace (Toyobo, Japan). The primers for RT-PCR were as follows: 5′-CGGAAGCATCGGACAT-3′ and 5′-CCTGACGAGCGAAACG-3′ for EcTSR, 5′-GTGCTGGAGAAAGAAGG-3′ and 5′-AACGCATCAGGTGAAAT-3′ for EcGCL, 5′-CTTCATAGGAATG GAAGCTGCGGGTA-3′ and 5-CGACCACCTTGATCTTCATGCTGCTA-3′ for rice *β-actin* gene (Os03g0718100).

### Western Blot analysis and enzyme assays

Proteins (crude extract) from rice plants were extracted by homogenizing 100 mg fresh leaves in 1 mL PBS (pH 8.0) and centrifuged at 12000 rpm for 10 min at 4 °C to remove cell debris. Proteins from protoplasts were extracted by adding 20 μL 250 mM Tris-HCl (pH6.8) containing 10% SDS, 5% β-mercaptoethanol to 80 μL of transform protoplasts (about 1.6 × 10^5^ cells), then incubated for 10 min at 95 °C and centrifuged at 12000 rpm for 10 min to remove cell debris. Equally loaded proteins (20 μg) were separated by SDS/PAGE. The GFP fusion proteins were probed by using a monoclonal anti-GFP antibody (Abmart, USA). The EcTSR or EcGCL protein from transgenic rice was probed by using a rabbit polyclonal antibody for EcTSR or EcGCL as described above, respectively. The β-actin protein was probed by using a monoclonal β-actin antibody (Abmart, USA) as loading control. Alkaline phosphatase antibody (Abmart, USA) was used as secondary antibody to detect the immunoreactive protein signals.

The EcTSR or EcGCL activity was assayed as described previously^48^. For EcTSR, reaction was initiated by the addition of 20 μL crude extract into 980 μL reaction mixture which consisted of 50 mM PBS (pH 8.0), 0.4 mM NADH, 5 mM tartronate semialdehyde. For EcGCL, reaction was initiated by the addition of 20 μL crude extract into 980 μL reaction mixture which consisted of 50 mM PBS (pH 8.0), 0.4 mM NADH, 0.4 mM thiamine hydrochloride, 8 mM MgCl_2_, 5 mM glyoxylate, 1 μg purified EcTSR protein. Both reactions were monitored at 340 nm using a spectrophotometer (Eppendorf BioSpectrometerskinetic) at 37 °C.

### Determination of the chloroplastic targeting efficiency

The targeting efficiency was determined by counting protoplasts based on the GFP fluorescence pattern of transformed protoplasts. The GFP pattern of a single protoplast could be summarized to four types observed by confocal laser microscopy (Supplemental Fig. S8): (i) totally in the chloroplasts; (ii) evenly distributed in both cytoplasm and chloroplasts; (iii) mostly localized to the cytoplasm; or (iv) totally in the cytoplasm. We defined these four types, respectively, as 100%, 50%, 10% and 0% targeting efficiency in a single cell. More than 200 protoplasts were counted each time and three identical experiments were performed to calculate the targeting efficiency for a certain target protein. The formula is: Targeting efficiency (%) = (100 × N_Type i_ + 50 × N_Type ii_ + 10 × N_Type iii_)/N_Total_, N represents the number of the counted cells. The counting data was listed in Supplemental Table S4.

### Protein tertiary structure analysis

The secondary and tertiary structure of EcTSR, OsCAT, CmMS, OsICL and OsOXO was predicted by SWISS-MODEL (http://swissmodel.expasy.org/). The tertiary structure of EPSP (UniProt ID: P0A6D3), NPT-II (UniProt ID: P00552), cry1Ac (UniProt ID: P05068), EcKAT (UniProt ID: P21179), EcGCL (UniProt ID: P0AEP7), OsrbcS (UniProt ID: Q01NY7) and the 80 precursor proteins were obtained from the RCSB protein database (http://www.rcsb.org/). The tertiary structure of HsI27 was described previously^40^.

## Additional Information

**How to cite this article:** Shen, B.-R. *et al*. An optimized transit peptide for effective targeting of diverse foreign proteins into chloroplasts in rice. *Sci. Rep.* **7**, 46231; doi: 10.1038/srep46231 (2017).

**Publisher's note:** Springer Nature remains neutral with regard to jurisdictional claims in published maps and institutional affiliations.

## Supplementary Material

Supplemental Data

## Figures and Tables

**Table 1:** The targeting efficiency of various foreign proteins in relation to the amino acid number of the N-terminus unfolded region.

Protein	Organism	N-term unfolded AA No.	Targeting efficiency (%)
EcTSR	*Escherichia coli*	1	4.3 ± 0.9
EcGCL	*Escherichia coli*	1	11.7 ± 0.9
HsI27	*Homo sapiens*	3	1.9 ± 0.7
OsICL	*Oryza sativa*	8	31.1 ± 5.7
OsCAT2	*Oryza sativa*	19	75.2 ± 4.1
CmMS	*Cucurbita moschata*	24	64.4 ± 4.4
OsOXO3	*Oryza sativa*	26	75.3 ± 5.1
EcKAT	*Escherichia coli*	52	83.9 ± 3.7
