# Supplementary material for: An optimized transit peptide for effective targeting of diverse foreign proteins into chloroplasts in rice
Source: Sci Rep. 2017 Apr 11;7:46231. doi: 10.1038/srep46231 (PMC5387683; doi:10.1038/srep46231)
Supplement: Supplemental Data [file srep46231-s1.pdf]

An optimized transit peptide for effective targeting of diverse foreign proteins into chloroplasts in rice

Bo-Ran Shen[1], Cheng-Hua Zhu[1], Zhen Yao[1], Li-Li Cui[1], Jian-Jun Zhang[1], Cheng-Wei Yang[2], Zheng-Hui He[3], Xin-Xiang Peng[1,] *

Supplemental Table S1

Supplemental Table S1. The length of transit peptides and N-terminal unfolded regions of the 80 precursor proteins collected from the PDB database.

protein	UniProt ID	Organism	Transit peptide AA No.	N-term unfolded AA No
Rubisco activase	P10896	*Arabidopsis thaliana*	58	8
Rubisco small subunit	P10796	*Arabidopsis thaliana*	55	21
Acetolactate synthase	P17597	*Arabidopsis thaliana*	55	41
Chlorophyll a/b binding protein	Q01667	*Arabidopsis thaliana*	35	37
Triosephosphate isomerase	Q9SKP6	*Arabidopsis thaliana*	60	5
Cysteine desulfurase I	Q93WX6	*Arabidopsis thaliana*	35	17
Glutamyl-tRNA reductase	P42804	*Arabidopsis thaliana*	64	30
Organellar oligopeptidase A	Q94AM1	*Arabidopsis thaliana*	82	8
Porphobilinogen deaminase	Q43316	*Arabidopsis thaliana*	62	11
CBS domain-containing protein	O23193	*Arabidopsis thaliana*	71	5
Adenylyl-sulfate kinase	Q43295	*Arabidopsis thaliana*	38	49
HTPA synthase II	Q9FVC8	*Arabidopsis thaliana*	39	18
MEP cytidylyltransferase	P69834	*Arabidopsis thaliana*	61	17
Shikimate kinase	Q8GY88	*Arabidopsis thaliana*	58	23
Beta-IPM dehydrogenase II	P93832	*Arabidopsis thaliana*	33	8
RCC reductase	Q8LDU4	*Arabidopsis thaliana*	39	12
Monothiol glutaredoxin	Q84Y95	*Arabidopsis thaliana*	63	4
DAP-aminotransferase	Q93ZN9	*Arabidopsis thaliana*	36	26
Peptide deformylase	Q9FUZ2	*Arabidopsis thaliana*	56	32
NifU-like protein	Q93W20	*Arabidopsis thaliana*	16	70
Cell division protein FtsY homolog	O80842	*Arabidopsis thaliana*	40	25
UDP-sulfoquinovose synthase	O48917	*Arabidopsis thaliana*	86	0

Threonine synthase	Q9S7B5	*Arabidopsis thaliana*	40	19
Allene oxide synthase	Q96242	*Arabidopsis thaliana*	21	44
Clp protease-related protein	Q8GW78	*Arabidopsis thaliana*	58	34
Phosphomethylpyrimidine synthase	O82392	*Arabidopsis thaliana*	37	50
Allene oxide cyclase	Q9LS02	*Arabidopsis thaliana*	77	4
Imidazoleglycerol-phosphate dehydratase	O23346	*Arabidopsis thaliana*	52	24
Chorismate mutase I	P42738	*Arabidopsis thaliana*	58	29
Single-stranded DNA-binding protein	Q9M9S3	*Arabidopsis thaliana*	47	37
Ent-copalyl diphosphate synthase	Q38802	*Arabidopsis thaliana*	60	31
CBS domain-containing protein	Q9C5D0	*Arabidopsis thaliana*	71	7
Phosphoglucan phosphatase	Q9SRK5	*Arabidopsis thaliana*	61	25
Bifunctional monothiol glutaredoxin-S16	Q8H7F6	*Arabidopsis thaliana*	62	10
Glyceraldehyde-3-phosphate dehydrogenase	P25856	*Arabidopsis thaliana*	60	1
Glutaredoxin-C5	Q8GWS0	*Arabidopsis thaliana*	61	6
Diaminopimelate epimerase	Q9LFG2	*Arabidopsis thaliana*	51	14
Haloacid dehalogenase-like hydrolase domain-containing protein	Q94K71	*Arabidopsis thaliana*	65	12
Oxygen-evolving enhancer protein II	P12302	*Spinacia oleracea*	81	18
Thioredoxin F-type	P09856	*Spinacia oleracea*	77	7
Rubisco small subunit	Q43832	*Spinacia oleracea*	57	21
Glyceraldehyde-3-phosphate dehydrogenase A	P19866	*Spinacia oleracea*	65	1
Thioredoxin M-type	P07591	*Spinacia oleracea*	67	13
Fructose-1,6-bisphosphatase	P22418	*Spinacia oleracea*	57	20
Photosystem I reaction center subunit III	P12355	*Spinacia oleracea*	77	5
Photosystem I reaction center subunit V	P12357	*Spinacia oleracea*	69	3
Ketol-acid reductoisomerase	Q01292	*Spinacia oleracea*	72	23
Photosystem I reaction center subunit XI	Q41385	*Spinacia oleracea*	47	49
Chlorophyll a-b binding protein	P12333	*Spinacia oleracea*	35	55
Photosystem II 22 kDa protein	Q02060	*Spinacia oleracea*	69	26
Stearoyl-ACP desaturase	P22337	*Spinacia oleracea*	33	33
Acyl carrier protein	P07854	*Spinacia oleracea*	56	2
Thioredoxin F-type	P09856	*Spinacia oleracea*	77	12

Ferredoxin--nitrite reductase	P05314	*Spinacia oleracea*	32	26
Ferredoxin-I	P00221	*Spinacia oleracea*	50	2
Oxygen-evolving enhancer protein III	P12301	*Spinacia oleracea*	83	4
Ferredoxin--NADP reductase	P10933	*Pisum sativum*	52	31
Chlorophyll a-b binding protein	P07371	*Pisum sativum*	37	57
Rubisco methyltransferase	Q43088	*Pisum sativum*	37	14
Fructose-1,6-bisphosphatase	P46275	*Pisum sativum*	50	20
Rubisco small subunit	P07689	*Pisum sativum*	57	21
Rubisco activase	Q40460	*Nicotiana tabacum*	58	12
Rubisco small subunit	P69249	*Nicotiana tabacum*	57	21
Oxygen-evolving enhancer protein	P18212	*Nicotiana tabacum*	79	17
Leucine aminopeptidase I	Q10712	*Solanum lycopersicum*	53	19
Threonine dehydratase biosynthetic	P25306	*Solanum lycopersicum*	51	13
Superoxide dismutase	P14831	*Solanum lycopersicum*	63	2
3-oxoacyl-[acyl-carrier-protein] reductase	Q93X62	*Brassica napus*	60	18
NADH-dependent enoyl-ACP reductase	P80030	*Brassica napus*	73	20
Protein CutA I	Q109R6	*Oryza sativa*	60	13
1,4-alpha-glucan-branching enzyme	Q01401	*Oryza sativa*	64	14
Granule-bound starch synthase I	Q0DEV5	*Oryza sativa*	77	6
Rubisco small subunit	Q0INY7	*Oryza sativa*	48	21
NifU-like protein	Q84LK7	*Oryza sativa*	76	2
Ketol-acid reductoisomerase	Q65XK0	*Oryza sativa*	52	25
Beta-D-glucoside glucohydrolase	P49235	*Zea mays*	54	28
Ferredoxin	P27787	*Zea mays*	52	1
Indole-3-glycerol phosphate lyase	P42390	*Zea mays*	53	36
Chloroplastic RNA splicing factor 2	Q9M5P4	*Zea mays*	45	16
9-cis-epoxycarotenoid dioxygenase I	O24592	*Zea mays*	23	63

Supplemental Table S2

Supplemental Table S2. The primers and the templates used for PCR.

Gene or sequence	GenBank ID	Preimer sequence	Templates	Comment
EcTSR	WP_021571777.1	5'- ATGAAACTGGGATTTA –3'<br>5'- TTAGGCCAGTTTATGG-3'	*E.coli* DNA	Completely length of ORF
EcGCL	WP_061352092.1	5'- ATGGCAAAAATGAGAG-3<br>5'- TTATTCATAGTGCATG -3	*E.coli* DNA	Completely length of ORF
EcKAT	WP_000077872.1	5'- ATGTCGCAACATAACG-3'<br>5'-TTAGGCAGGAATTTTG-3'	*E.coli* DNA	Completely length of ORF
OsCAT	Os03g0131200	5'-ATGGATCCCTACAAGC-3'<br>5'-TTACGCCAGTTTCTGA-3'	Rice leave cDNA	The last 9 amino acids on the C-term were removed
OsOXO	Os03g0693900	5'- ATGGACCCTCTGCAGG-3'<br>5'- TTAGTACCCGCCGGTG-3'	Rice leave cDNA	Completely length of ORF
OsICL	Os07g0529000	5'- ATGTCGTCGCCGTTCT-3'<br>5'- TTTGGCAAGAACATGG-3'	Rice leave cDNA	The last 3 amino acids on the C-term were removed
OsRbcS	Os12g0274700	5'- ATGGCCCCCTCCGTGT-3'<br>5'- TTAGTTGCCACCAGAC-3	Rice leave cDNA	Completely length of ORF
rCTP	Os12g0274700	5'- ATGGCCCCCTCCGTGT-3'<br>5'-CATGCACCTGATCCTG-3'	Rice leave cDNA	
CmMS	CAA40262.1	5'- ATGGGATCGCTGGGAT-3'<br>5'- CAGCTCCCTGGGATGA-3'	Pumpkin leave cDNA	The last 3 amino acids on the C-term were removed
HsI27	NP_001254479.2	5'- CGACTAATAAAAGTGG-3'<br>5'- CAATTCTTTCACTTTC-3'		
GFP	AAF62891.1	5'- ATGGTGAGCAAGGGCG-3'<br>5'- TTACTTGTACAGCTCG-3'	Cloning vector GFP-pUC18	
aCTP	AT1G67090	5'- ATGGCTTCCTCTATGC-3'<br>5'- CATGCAGTTAACTCTT-3'	Arabidopsis leave cDNA	
aCTP-N21	AT1G67090	5'- ATGGCTTCCTCTATGC-3'<br>5'-GGTAAGGTCAGGAAGG-3	Arabidopsis leave cDNA	
tCTP	AFB70994.1	5'- ATGGCTTCCTCAGTTC-3'<br>5'- CATGCATTGCACTCTT-3'	Tobacco leave cDNA	
tCTP-N21	AFB70994.1	5'- ATGGCTTCCTCAGTTC-3'<br>5'- ATGCACCTAACTCTTC-3'	Tobacco leave cDNA	
rCTP-N21	Os12g0274700	5'- ATGGCCCCCTCCGTGT-3'<br>5'-GAGCGGTGGCAGGTAG-3	*OsRbcS* sequence	
RbcS folding region (FR)	Os12g0274700	5'-ACCGTGGAGGACCTCC-3'<br>5'-GTTGCCACCAGACTCC-3	*OsRbcS* sequence	
rCTP-5A		5'- ATGGCTTCCTCAGTTC-3'<br>5'-CGCTGCAGCGGCTGCCATGCACCTGATCCTG-3'	*OsRbcS* sequence	Addition of a 5-alanine sequence to rCTP C-term

rCTP-10A	5'- ATGGCTTCCTCAGTTC-3'<br>5'-TGCAGCGGCTGCCGCCGCTGCA<br>GCGGCTGCCATGCACCTGATCCTG-<br>3'	*OsRbcS* sequence	Addition of a 10-alanine sequence to rCTP C-term
rCTP-15A	5'- ATGGCTTCCTCAGTTC-3'<br>5'-CGCTGCAGCGGCTGCTGCAGCG<br>GCTGCCGCCGCTGCAGCGGCTGCC<br>ATGCACCTGATCCTG-3'	*OsRbcS* sequence	Addition of a 15-alanine sequence to rCTP C-term
rCTP-20A	5'- ATGGCTTCCTCAGTTC-3'<br>5'-TGCAGCGGCTGCCGCCGCTGCA<br>GCGGCTGCTGCAGCGGCTGCCGCC<br>GCTGCAGCGGCTGCCATGCACCTG<br>ATCCTG-3'	*OsRbcS* sequence	Addition of a 20-alanine sequence to rCTP C-term
rCTP-25A	5'- ATGGCTTCCTCAGTTC-3'<br>5'-CGCTGCAGCGGCTGCTGCAGCG<br>GCTGCCGCCGCTGCAGCGGCTGCT<br>GCAGCGGCTGCCGCCGCTGCAGC<br>GGCTGCCATGCACCTGATCCTG-3'	*OsRbcS* sequence	Addition of a 25-alanine sequence to rCTP C-term
rCTP-N21(T1)	5'- ATGGCTTCCTCAGTTC-3'<br>5'-GAGCGGTGGCAGGTAGGAGAG<br>GGTCTCGAACTTCTTGATGCCCTC<br>AGCCGCTGCAGCTGCCATGCACCT<br>GATCCTG-3'	*OsRbcS* sequence	Generation the T1 mutant of N21
rCTP-N21(T2)	5'- ATGGCTTCCTCAGTTC-3'<br>5'-GAGCGGTGGCAGGTAGGAGAG<br>GGTCTCGAAAGCCGCTGCAGCTGC<br>AATCGGCCACACCTGC-3'	*OsRbcS* sequence	Generation the T2 mutant of N21
rCTP-N21(T3)	5'- ATGGCTTCCTCAGTTC-3'<br>5'-GAGCGGTGGCAGGTAAGCCGCT<br>GCAGCTGCCTTCTTGATGCCCTCA-<br>3'	*OsRbcS* sequence	Generation the T3 mutant of N21
rCTP-N21(T4)	5'- ATGGCTTCCTCAGTTC-3'<br>5'-AGCCGCTGCAGCTGCGGAGAGG<br>GTCTCGAAC-3'	*OsRbcS* sequence	Generation the T4 mutant of N21
rCTP-N21(D1)	5'- ATGGCTTCCTCAGTTC-3'<br>5'-GAGCGGTGGCAGGTAGGAGAG<br>GGTCTCGAACTTCTTGATGCCCTC<br>CATGCACCTGATCCTG-3'	*OsRbcS* sequence	Generation the D1 mutant of N21
rCTP-N21(D2)	5'- ATGGCTTCCTCAGTTC-3'<br>5'-GAGCGGTGGCAGGTAGGAGAG<br>GGTCTCGAAAATCGGCCACACCTG<br>C-3'	*OsRbcS* sequence	Generation the D2 mutant of N21
rCTP-N21(D3)	5'- ATGGCTTCCTCAGTTC-3'<br>5'-GAGCGGTGGCAGGTACTTCTTG	*OsRbcS* sequence	Generation the D3 mutant of N21

	ATGCCCTCA-3’		
rCTP-N21(D4)	5’- ATGGCTTCCTCAGTTC-3’<br>5’- GGAGAGGGTCTCGAAC-3	*OsRbcS* sequence	Generation the D4 mutant of N21
rCTP-N21-BS1 (RC1)	5’- ATGGCTTCCTCAGTTC-3’<br>5’-GGAGGCCTTGATTGACATCCTG GGGGTGGCGACGGCGACCTTGAC GGTGAGCGGTGGCAGGTAG-3’	*OsRbcS* sequence	Generation the RC1 sequence
rCTP-N21-BS2 (RC2)	5’- ATGGCTTCCTCAGTTC-3’<br>5’-ACATCGTATTCTCCCTCCGTTGG ACACATTTCCGAAGCTGCTATTTCC GAGCGGTGGCAGGTAG-3’	*OsRbcS* sequence	Generation the RC2 sequence
EcTSR Part a	5’-ATGAAACTGGGATTTA-3’<br>5’-GTTACCGCCCACGAGG-3	*EcTSR* sequence	From amino acids 1 to 156
EcTSR Part b	5’- GGCGATGGTCAAACCT-3’<br>5’- GGCCAGTTTATGGTTA-3’	*EcTSR* sequence	From amino acids 157 to 293
EcTSR Part c	5’- CAGGTTGAAGAAGTTC-3’<br>5.- CACACGTACCGGGTCC-3’	*EcTSR* sequence	From amino acids 100 to 200
EcTSR Part a1	5’-ATGAAACTGGGATTTA-3’<br>5’- GGTGTGTCCGGCACCA-3’	*EcTSR* sequence	From amino acids 1 to 67
EcTSR Part a2	5’- CAGGTTGAAGAAGTTC-3’<br>5’- GTTACCGCCCACGAGG-3’	*EcTSR* sequence	From amino acids 68 to 156
EcTSR Part a3	5’- GCTGATGAATTACTGT-3’<br>5’- AGTTTCAATCGGGGAA-3’	*EcTSR* sequence	From amino acids 34 to 95

Supplemental Table S3

Supplemental Table S3. The restriction enzyme sequences used for generating the fusion genes.

Fusion gene	Sequence for genes fusion
rCTP-GFP	rCTP – *Sac* I GAGCTC – GFP
aCTP-GFP	aCTP – *Sac* I GAGCTC – GFP
tCTP-GFP	tCTP – *Sac* I GAGCTC – GFP
rCTP-N21-GFP	rCTP – N21 – *Sac* I GAGCTC – GFP
aCTP-N21-GFP	aCTP – N21 – *Sac* I GAGCTC – GFP
tCTP-N21-GFP	tCTP – N21 – *Sac* I GAGCTC – GFP
rCTP-EcTSR-GFP	rCTP – *Hind* III AAGCTT – EcTSR – *Bam*H I GGATCC – GFP
aCTP-EcTSR-GFP	aCTP – *Hind* III AAGCTT – EcTSR – *Bam*H I GGATCC – GFP
tCTP-EcTSR-GFP	tCTP – *Hind* III AAGCTT – EcTSR – *Bam*H I GGATCC – GFP
rCTP-N21-EcTSR-GFP	rCTP – N21 – *Hind* III AAGCTT – EcTSR – *Bam*H I GGATCC – GFP
aCTP-N21-EcTSR-GFP	aCTP – N21 – *Hind* III AAGCTT – EcTSR – *Bam*H I GGATCC – GFP
tCTP-N21-EcTSR-GFP	tCTP – N21 – *Hind* III AAGCTT – EcTSR – *Bam*H I GGATCC – GFP

rCTP-EcGCL-GFP	rCTP · *Hind* III AAGCTT · EcGCL · *Bam*H I GGATCC · GFP
rCTP-OsCAT-GFP	rCTP · *Hind* III AAGCTT · OsCAT · *Bam*H I GGATCC · GFP
rCTP-OsICL-GFP	rCTP · *Hind* III AAGCTT · OsICL · *Bam*H I GGATCC · GFP
rCTP-OsOXO-GFP	rCTP · *Hind* III AAGCTT · OsOXO · *Bam*H I GGATCC · GFP
rCTP-CmMS-GFP	rCTP · *Hind* III AAGCTT · CmMS · *Bam*H I GGATCC · GFP
rCTP-HsI27-GFP	rCTP · *Hind* III AAGCTT · HsI27 · *Bam*H I GGATCC · GFP
rCTP-EcTSR$_{part\ a}$-GFP	rCTP · *Hind* III AAGCTT · Part a · *Bam*H I GGATCC · GFP
rCTP-EcTSR$_{part\ b}$-GFP	rCTP · *Hind* III AAGCTT · Part b · *Bam*H I GGATCC · GFP
rCTP-EcTSR$_{part\ c}$-GFP	rCTP · *Hind* III AAGCTT · Part c · *Bam*H I GGATCC · GFP
rCTP-EcTSR$_{part\ a1}$-GFP	rCTP · *Hind* III AAGCTT · Part a1 · *Bam*H I GGATCC · GFP
rCTP-EcTSR$_{part\ a2}$-GFP	rCTP · *Hind* III AAGCTT · Part a2 · *Bam*H I GGATCC · GFP
rCTP-EcTSR$_{part\ a3}$-GFP	rCTP · *Hind* III AAGCTT · Part a3 · *Bam*H I GGATCC · GFP
rCTP-GFP-EcTSR	tCTP · *Sac* I GAGCTC · GFP · *Hind* III AAGCTT · EcTSR
rCTP-5A-EcTSR-GFP	rCTP · 5A · *Hind* III AAGCTT · EcTSR · *Bam*H I GGATCC · GFP

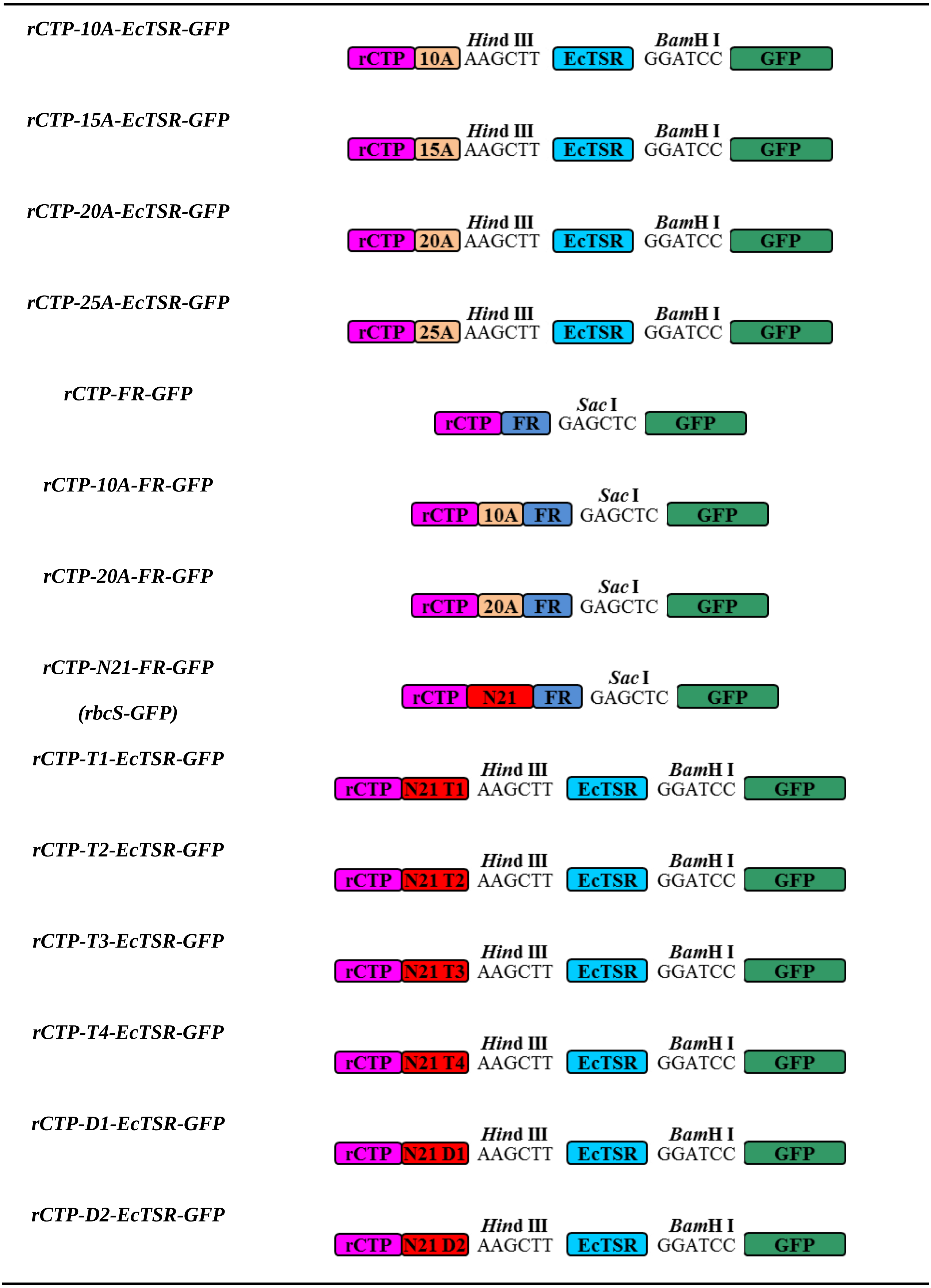
rCTP-10A-EcTSR-GFP
rCTP 10A Hind III AAGCTT EcTSR BamH I GGATCC GFP
rCTP-15A-EcTSR-GFP
rCTP 15A Hind III AAGCTT EcTSR BamH I GGATCC GFP
rCTP-20A-EcTSR-GFP
rCTP 20A Hind III AAGCTT EcTSR BamH I GGATCC GFP
rCTP-25A-EcTSR-GFP
rCTP 25A Hind III AAGCTT EcTSR BamH I GGATCC GFP
rCTP-FR-GFP
rCTP FR Sac I GAGCTC GFP
rCTP-10A-FR-GFP
rCTP 10A FR Sac I GAGCTC GFP
rCTP-20A-FR-GFP
rCTP 20A FR Sac I GAGCTC GFP
rCTP-N21-FR-GFP
(rbcS-GFP)
rCTP N21 FR Sac I GAGCTC GFP
rCTP-T1-EcTSR-GFP
rCTP N21 T1 Hind III AAGCTT EcTSR BamH I GGATCC GFP
rCTP-T2-EcTSR-GFP
rCTP N21 T2 Hind III AAGCTT EcTSR BamH I GGATCC GFP
rCTP-T3-EcTSR-GFP
rCTP N21 T3 Hind III AAGCTT EcTSR BamH I GGATCC GFP
rCTP-T4-EcTSR-GFP
rCTP N21 T4 Hind III AAGCTT EcTSR BamH I GGATCC GFP
rCTP-D1-EcTSR-GFP
rCTP N21 D1 Hind III AAGCTT EcTSR BamH I GGATCC GFP
rCTP-D2-EcTSR-GFP
rCTP N21 D2 Hind III AAGCTT EcTSR BamH I GGATCC GFP

rCTP-D3-EcTSR-GFP

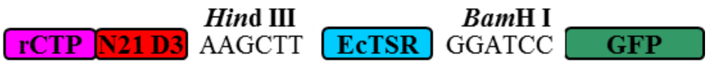

rCTP-D4-EcTSR-GFP

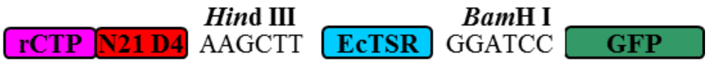

rCTP-N21-BS1-GFP

(RC1-GFP)

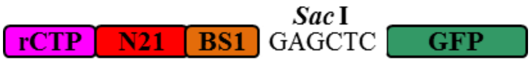

rCTP-N21-BS2-GFP

(RC2-GFP)

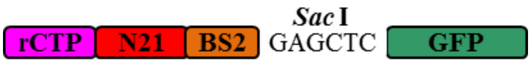

RC2-EcTSR-GFP

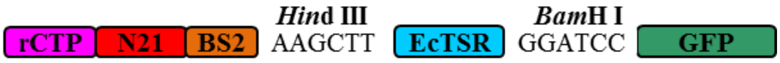

RC2-EcGCL-GFP

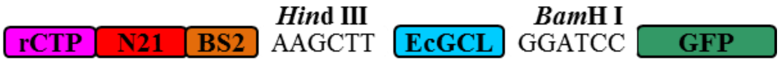

RC2-HsI27-GFP

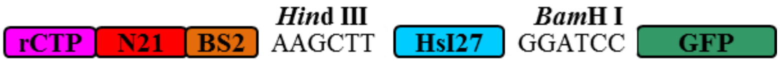

RC2-OsICL-GFP

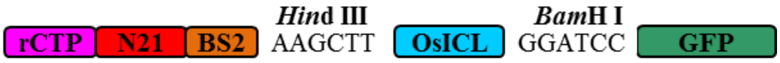

Supplemental Table S4

Supplemental Table S4. Counting data for calculating the targeting efficiency of fusion proteins. Targeting efficiency (%) = (100×NType i + 50×NType ii + 10×NType iii) / NTotal, N represents the number of the counted cells. The data represent means ±SD of three biological replicates (*n*=3).

Fusion gene	Repeat 1				Repeat 2				Repeat 3				Efficiency %
	N_{Type} i	N_{Type} ii	N_{Type} iii	N_{Type} iv	N_{Type} i	N_{Type} ii	N_{Type} iii	N_{Type} iv	N_{Type} i	N_{Type} ii	N_{Type} iii	N_{Type} iv	
rCTP-EcTSR-GFP	0	18	0	190	0	22	2	192	0	14	0	190	4.3±0.9
rCTP-EcGCL-GFP	0	20	170	42	0	22	168	64	0	24	176	40	11.7±0.9
rCTP-HsI27-GFP	0	6	4	220	0	12	2	218	0	6	4	230	1.9±0.7
rCTP-OsICL-GFP	2	108	62	46	1	92	67	34	5	158	39	30	31.1±5.7
rCTP-OsCAT-GFP	156	118	8	0	118	150	6	0	178	96	16	0	75.2±4.1
rCTP-OsOXO-GFP	138	72	8	0	100	144	2	0	128	106	6	0	75.3±5.1
rCTP-CmMS-GFP	118	120	12	10	96	96	22	14	104	116	38	8	64.4±4.4
rCTP-EcKAT-GFP	138	66	4	0	154	48	0	0	136	68	6	0	83.9±3.7
rCTP-5A-EcTSR-GFP	10	102	24	94	14	114	12	94	4	110	16	102	28.2±2.4
rCTP-10A-EcTSR-GFP	28	124	14	56	22	120	10	62	26	148	6	42	41.8±3.3
rCTP-15A-EcTSR-GFP	54	136	6	34	48	122	18	38	58	150	8	40	51.5±2.2
rCTP-20A-EcTSR-GFP	92	102	10	28	86	114	22	20	128	118	16	36	62.0±2.0
rCTP-25A-EcTSR-GFP	106	100	18	34	110	94	26	30	98	98	14	26	61.8±0.9
rCTP-GFP	164	92	8	0	136	98	4	0	154	100	14	0	78.1±1.6
rCTP-N21-GFP	158	56	4	0	184	52	0	0	176	62	10	0	86.1±2.6
rCTP-N21-FR-GFP	172	48	10	0	188	50	6	0	166	58	10	0	85.7±1.9
rCTP-FR-GFP	10	100	46	110	20	88	40	92	10	124	64	64	27.5±2.9
rCTP-10A-FR-GFP	56	138	38	44	52	150	54	46	60	118	46	34	46.1±2.1
rCTP-20A-FR-GFP	112	128	22	26	108	114	26	22	118	104	16	18	63.6±2.9
rCTP-N21-EcTSR-GFP	160	60	6	0	163	78	2	0	172	54	0	0	85.3±2.5

rCTP-T1-EcTSR-GFP	154	58	8	0	164	50	2	0	158	62	2	0	84.4 ±2.9
rCTP-T2-EcTSR-GFP	60	128	14	12	92	110	6	18	96	140	12	20	62.1 ±3.4
rCTP-T3-EcTSR-GFP	140	64	8	2	136	68	6	9	144	90	18	6	76.4 ±4.1
rCTP-T4-EcTSR-GFP	156	60	6	0	164	48	12	0	174	54	2	2	85.1 ±1.4
rCTP-D1-EcTSR-GFP	64	196	8	8	52	162	14	12	44	184	16	8	56.5 ±2.2
rCTP-D2-EcTSR-GFP	42	156	24	46	38	192	14	32	54	168	10	44	48.4 ±2.4
rCTP-D3-EcTSR-GFP	68	172	10	0	58	156	8	12	40	146	14	8	58.5 ±3.5
rCTP-D4-EcTSR-GFP	52	164	26	8	40	148	14	12	56	158	20	10	54.6 ±1.5
aCTP-N21-GFP	114	156	6	0	140	144	2	0	96	140	4	0	71.0 ±2.5
tCTP-N21-GFP	134	160	0	0	132	176	6	0	140	140	4	0	72.0 ±2.5
aCTP-N21-EcTSR-GFP	114	180	12	4	126	176	6	0	88	178	26	0	65.8 ±4.1
tCTP-N21-EcTSR-GFP	84	188	10	0	98	176	6	0	96	180	14	0	64.9 ±1.6
RC1-GFP	172	78	10	0	192	50	6	0	202	52	2	0	86.4 ±3.3
RC2-GFP	166	60	6	0	162	78	2	0	186	22	0	0	87.3 ±6.4
RC2-EcTSR-GFP	146	58	8	4	158	62	6	1	172	50	2	0	84.2 ±3.4
RC2-EcGCL-GFP	172	54	18	0	178	52	12	1	164	34	4	0	85.4 ±3.9
RC2-HsI27-GFP	152	54	4	8	158	46	6	4	166	38	2	4	85.1 ±3.0
RC2-OsICL-GFP	178	32	10	2	158	62	18	4	164	44	4	0	84.6 ±5.0

Supplemental Figure S1

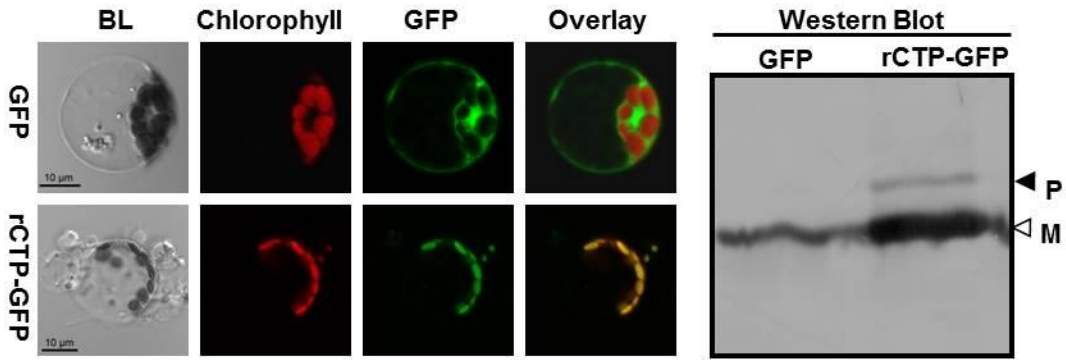

Supplemental Figure S1. Localization analyses of GFP and rCTP-GFP proteins in rice transgenic protoplasts.

Plasmids containing the *rCTP-GFP* fusion gene and *GFP* gene were introduced into rice protoplasts. Cells are imaged by a confocal microscope at 24h after the transfection. **BL**, bright light; **chlorophyll**, chloroplast chloroplyll autofluorescence; **GFP**, GFP fluorescence; Proteins extracted from the transfected protoplasts were analyzed by Western Blot using a monoclonal anti-GFP antibody. **P**, precursor form; **M**, mature form.

Supplemental Figure S2

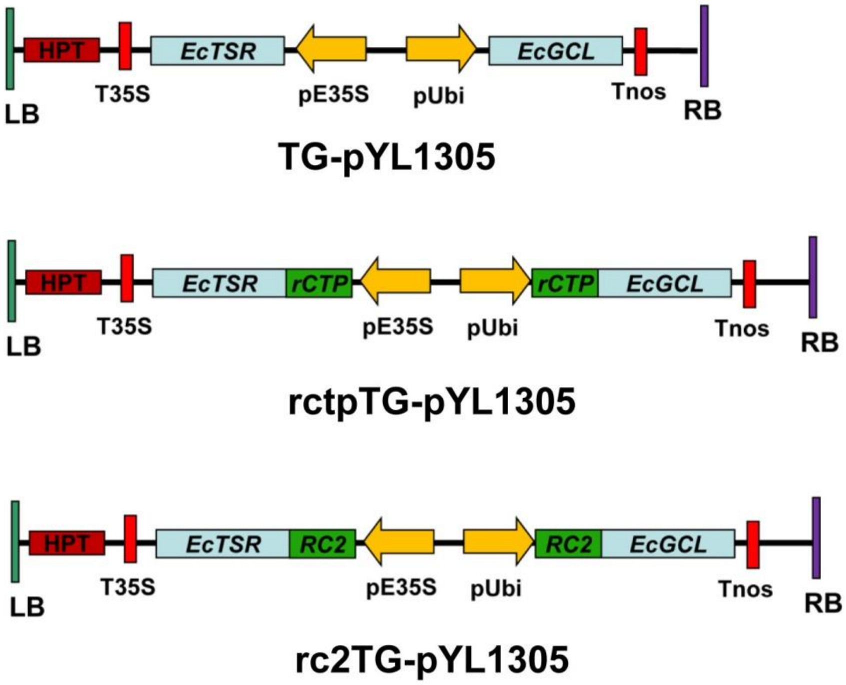

Supplemental Figure S2. Structure of the multiple genes expression vectors. **pE35S**, CaMV *35S* enhance promoter; **T35S**, CaMV *35S* terminator; **pUbi**, *ubi* promoter; **Tnos**, *nos* terminator; **HTP**, expression cassette of *hygromycin phosphotransferase* gene; **LB**, left border; **RB**, right border.

Supplemental Figure S3

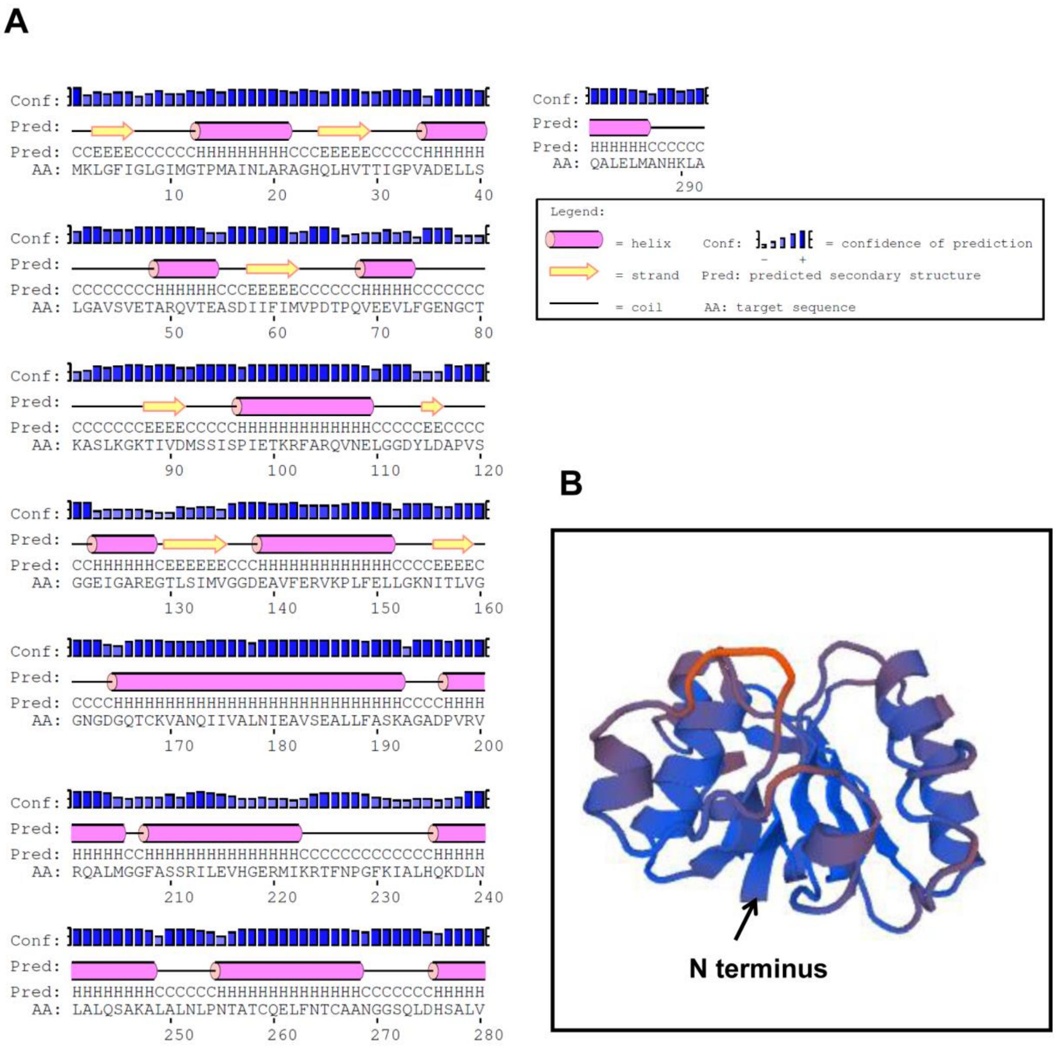

Supplemental Figure S3. Secondary and tertiary structure of EcTSR.

(A) Secondary structure of EcTSR.

(B) Tertiary structure of EcTSR first domain (F420 oxidored domain).

The secondary structure and tertiary structure of EcTSR were predicted by SWISS-MODLE using the amino sequence of EcTSR (WP_021571777.1).

Supplemental Figure S4

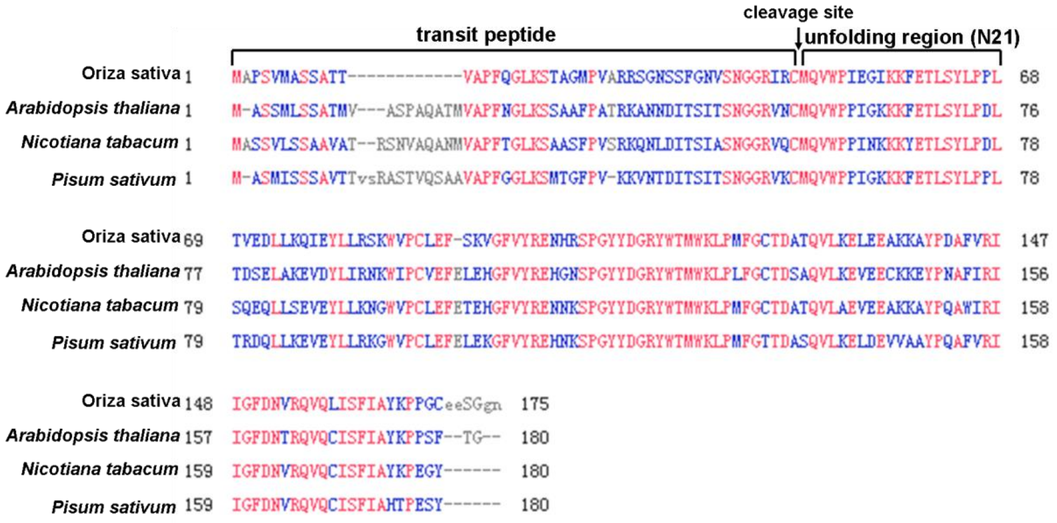

Supplemental Figure S4. Sequence alignment analysis of the rbcS from rice, Arabidopsis, tobacco and pea.

The amino acid sequence of the rbcS from rice (AAC14064.1), Arabidopsis (NP_176880.1), tobacco (CUA55116.1) and pea (P07689.1) was aligned through BLAST.

Supplemental Figure S5

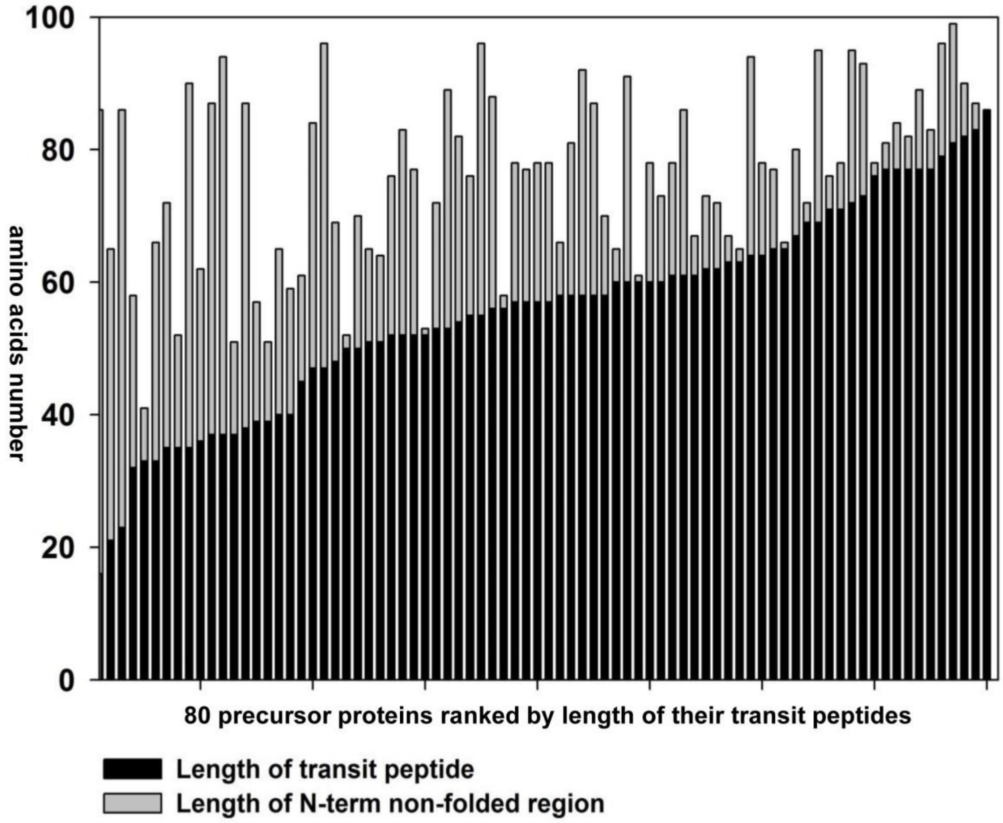

Supplemental Figure S5. The length of the transit peptides and N-terminal unfolded regions of the 80 precursor proteins.

The average length of the transit peptides is 55.7 amino acids, the average length of N-terminal unfolded regions is 20.3 amino acids.

Supplemental Figure S6

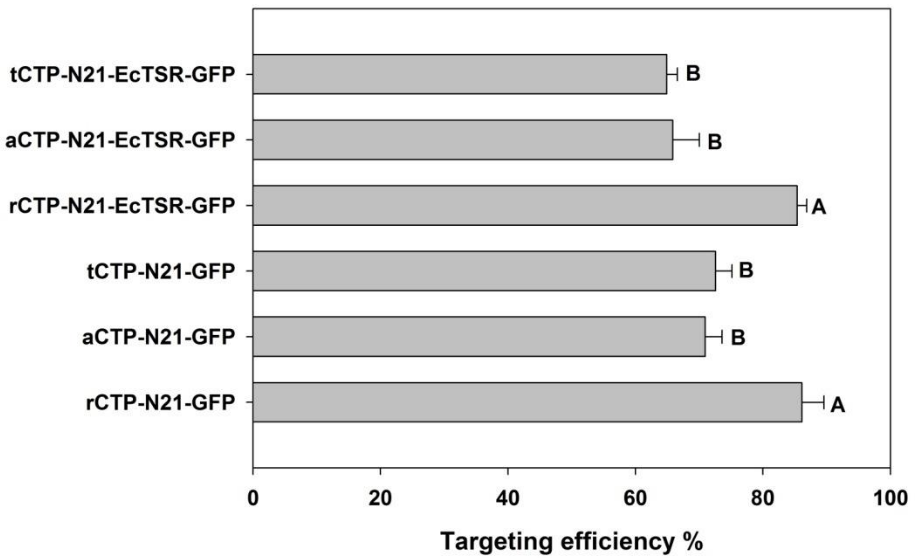

Supplemental Figure S6. Comparison of the chloroplast targeting efficiency of rbcS transit peptides from rice (rCTP), Arabidopsis (aCTP) and tobacco (tCTP). Plasmids containing the *tCTP-N21-EcTSR-GFP*, *tCTP-N21-EcTSR-GFP*, *tCTP-N21-EcTSR-GFP*, *tCTP-N21-GFP*, *aCTP-N21-GFP* or *rCTP-N21-GFP* fusion gene were introduced into rice protoplasts for targeting efficiency analysis. The data represent means ± SD of three biological replicates (*n*=3). Different capital letters in the same column indicate significant differences at P <0.01 according to Duncan's multiple range test.

Supplemental Figure S7

A

Thr-Val-Lys-Val-Ala-Val-Ala-Thr-Pro-Arg-Met-Ser-Ile-Lys-Ala-Ser ——BS1

Gly-Asn-Ser-Ser-Phe-Gly-Asn-Val-Ser-Asn-Gly-Gly-Arg-Ile-Arg-Cys ——BS2

B

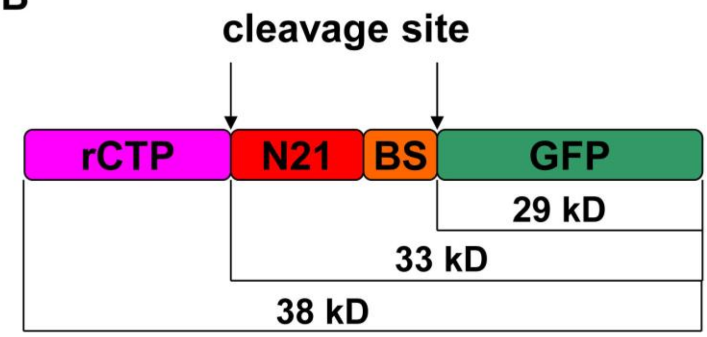

C

MAPSVMASSATTVAPFQGLKSTAGMPVARRSGNSSFGNVSNG

GRIRCMQVWPIEGIKKFETLSYLPPLGNSSFGNVSNGGRIRCM

N21 BS2

cleavage site cleavage site

D

ATGGCCCCCTCCGTGATGGCGTCGTCGGCCACCACCGTCGCT

CCCTTCCAGGGGCTCAAGTCCACCGCCGGCATGCCCGTCGCC

CGCCGCTCCGGCAACTCCAGCTTCGGCAACGTCAGCAATGGC

GGCAGGATCAGGTGCATGCAGGTGTGGCCGATTGAGGGCAT

CAAGAAGTTCGAGACCCTCTCCTACCTGCCACCGCTCGGAAA

TAGCAGCTTCGGAAATGTGTCCAACGGAGGGAGAATACGAT

GT

Supplemental Figure S7. The structure of the improved CTP.

(A) The amino acid sequences of SPP binding sites BS1 and BS2.

(B) The structure of the RC-GFP fusion protein. The RC sequences are comprised of rCTP, N21 and BS1 or BS2. The RC-GFP fusion proteins contain two cleavage sites, and the theoretical molecular weights of precursor and mature forms of RC-GFP are about 38 kD and 29 kD, respectively.

(C) The amino acid sequence of RC2.

(D) The nucleotide sequence of RC2.

Supplemental Figure S8

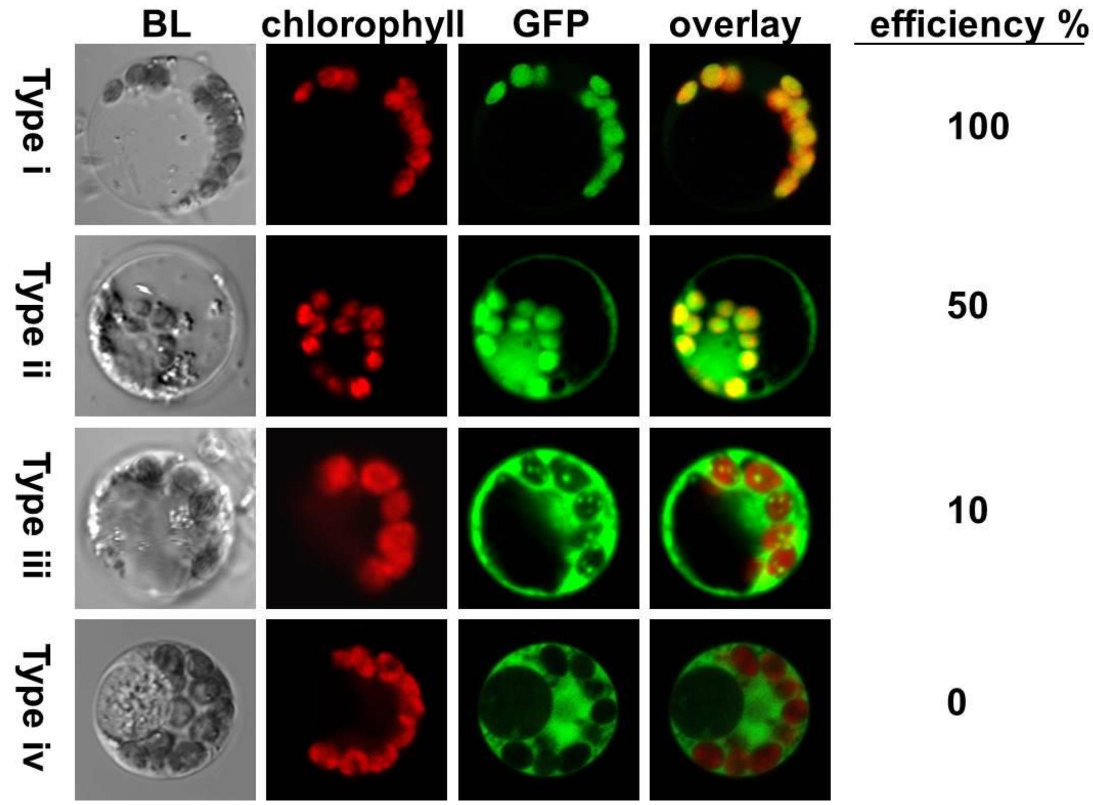

Supplemental Figure S8. GFP fluorescence pattern of transformed protoplasts.

The four types of GFP fluorescence pattern were imaged from *rCTP-EcTSR (part a1)-GFP*, *rCTP-OsICL-GFP*, *rCTP-EcGCL-GFP* or *rCTP-EcTSR (part a)-GFP* fusion gene transformed protoplasts respectively.
